# Transcriptome sequencing of two wild barley (*Hordeum spontaneum* L.) ecotypes differentially adapted to drought stress reveals ecotype-specific transcripts

**DOI:** 10.1186/1471-2164-15-995

**Published:** 2014-11-19

**Authors:** Girma Bedada, Anna Westerbergh, Thomas Müller, Eyal Galkin, Eyal Bdolach, Menachem Moshelion, Eyal Fridman, Karl J Schmid

**Affiliations:** Department of Plant Biology, Uppsala BioCenter, Linnean Centre of Plant Biology in Uppsala, Swedish University of Agricultural Sciences (SLU), Uppsala, Sweden; Institute of Plant Science and Genetics, The Hebrew University of Jerusalem, Rehovot, Israel; Institute for Plant Breeding, Seed Science and Population Genetics, University of Hohenheim, Fruwirthstrasse 21, D-70599 Stuttgart, Germany

**Keywords:** *Hordeum spontaneum*, Transcriptome, Drought tolerance, Genetic diversity

## Abstract

**Background:**

Wild barley is adapted to highly diverse environments throughout its geographical distribution range. Transcriptome sequencing of differentially adapted wild barley ecotypes from contrasting environments contributes to the identification of genes and genetic variation involved in abiotic stress tolerance and adaptation.

**Results:**

Two differentially adapted wild barley ecotypes from desert (B1K2) and Mediterranean (B1K30) environments were analyzed for drought stress response under controlled conditions. The desert ecotype lost more water under both irrigation and drought, but exhibited higher relative water content (RWC) and better water use efficiency (WUE) than the coastal ecotype. We sequenced normalized cDNA libraries from drought-stressed leaves of both ecotypes with the 454 platform to identify drought-related transcripts. Over half million reads per ecotype were *de novo* assembled into 20,439 putative unique transcripts (PUTs) for B1K2, 21,494 for B1K30 and 28,720 for the joint assembly. Over 50% of PUTs of each ecotype were not shared with the other ecotype. Furthermore, 16% (3,245) of B1K2 and 17% (3,674) of B1K30 transcripts did not show orthologous sequence hits in the other wild barley ecotype and cultivated barley, and are candidates of ecotype-specific transcripts. Over 800 unique transcripts from each ecotype homologous to over 30 different stress-related genes were identified. We extracted 1,017 high quality SNPs that differentiated the two ecotypes. The genetic distance between the desert ecotype and cultivated barley was 1.9-fold higher than between the Mediterranean ecotype and cultivated barley. Moreover, the desert ecotype harbored a larger proportion of non-synonymous SNPs than the Mediterranean ecotype suggesting different demographic histories of these ecotypes.

**Conclusions:**

The results indicate a strong physiological and genomic differentiation between the desert and Mediterranean wild barley ecotypes and a closer relationship of the Mediterranean to cultivated barley. A significant number of novel transcripts specific to wild barley were identified. The higher SNP density and larger proportion of SNPs with functional effects in the desert ecotype suggest different demographic histories and effects of natural selection in Mediterranean and desert wild barley. The data are a valuable genomic resource for an improved genome annotation, transcriptome studies of drought adaptation and a source of new genetic markers for future barley improvement.

**Electronic supplementary material:**

The online version of this article (doi:10.1186/1471-2164-15-995) contains supplementary material, which is available to authorized users.

## Background

Plants use different response and adaptive mechanisms to deal with abiotic water deficit stress [1–6]. These include (i) avoidance of dehydration by closing stomata to decrease water loss and to maintain turgor or osmotic pressure; (ii) drought escape by a rapid life cycle to cope with water deficit; and (iii) dehydration tolerance and maintenance of growth and development under low-water status by accumulating protective proteins, sugars, proline, antioxidants, by maintaining cell membrane stability, and by interrupting metabolic activity through dormancy. Abiotic stress response mechanisms in plants are linked to different physiological traits. Depending on their molecular and physiological attributes, plants control their stomatal aperture and water balance in very different ways with important consequences for their transpiration, biomass gain and survival. Accordingly, drought-tolerant plant ecotypes may have more flexible stomatal responses under drought conditions by sustaining longer periods of transpiration and CO_2_ assimilation. They may therefore outperform plants with more sensitive stomatal responses under conditions of mild to moderate drought.

Cultivated barley (*Hordeum vulgare* L.) is one of the most important global crops because it can be cultivated in highly diverse environments. Wild barley (*Hordeum spontaneum* L.) is the direct ancestor of cultivated barley and has a large geographical distribution ranging from deserts to highland climates [7]. Throughout its geographical distribution, wild barley is exposed to multiple environmental stresses such as drought, high temperatures, and high soil salinity. The correlation of genetic and environmental variation within its distribution range suggests the action of local adaptation along macro- and micro-environmental gradients [8–10]. It is therefore an excellent model system for studying plant adaptation and holds a high potential as a genetic resource for the breeding of stress tolerant varieties of cultivated barley [11, 12].

In barley, several traits characterize the differential response to drought stress, including grain carbon isotope discrimination [13], leaf relative water content [14], leaf osmotic potential and adjustment, and osmotic potential at full turgor [15, 16], water-soluble carbohydrate concentration [15] and chlorophyll parameters (e.g. fluorescence) [17]. These traits are correlated with morphological and phenological traits such as variation in seedling germination, flowering time, plant height and tillering, root growth and grain yield. Some of them differ between desert and Mediterranean wild barley ecotypes suggesting local adaptation [18–20]. So far, over 100 quantitative trait loci (QTLs) were found to be associated with drought stress responsive traits in cultivated barley [1, 13–16, 21, 22]. The complex nature of the trait and current shortcomings in QTL detection are responsible for the limited contribution of QTL studies to elucidate the genetic basis of drought tolerance and its utilization in plant breeding [2]. Modern genomic approaches may lead to a more efficient use of genetic resources like wild barley to utilize drought stress tolerance genes for crop improvement.

The recent sequencing of the barley genome greatly facilitates the mapping and utilization of useful genetic variation [23]. The current genome assembly is based on five cultivated barley cultivars and one wild barley accession of the Barley1K collection (B1K-04-12, hereafter called B1K4) [9], which resulted in 26,159 'high-confidence’ (HC) barley genes and a total estimate of 30,400 genes [23]. Levels of genome-wide variation were high in both wild and cultivated barley, but with an almost two-fold higher level of genetic variation in wild than in cultivated barley. This suggests that much untapped and potentially useful genetic variation is segregating in wild barley [24, 25]. Re-sequencing, transcriptome sequencing and sequencing of epigenomic variation contributes to the functional annotation of complex plant genomes and to the identification of genes and alleles responsible for adaptive evolution in contrasting environments [26, 27].

To identify genes expressed under drought stress in differentially adapted wild barley accessions, we first validated the different physiological adaptation to drought stress of two wild barley ecotypes from a collection of *Hordeum spontaneum* ecotypes (Barley1K) [9] and then performed 454 transcriptome sequencing of normalized cDNA libraries from drought-stressed leaves of these two accessions because transcriptome sequencing allow the identification of novel genes and improve the annotation of the barley genome. The two sequenced ecotypes included a desert ecotype (B1K-2-8, hereafter called B1K2) from the Negev desert, and a 'Mediterranean’ ecotype (B1K-30-09, hereafter called B1K30) from the cooler and moister North-Western region of Israel. Both genetic and phenotypic analyses strongly suggested that they are differentially adapted to heat and drought stress [20]. We identified numerous ecotype-specific genes, which may be involved in drought adaptation, and single nucleotide polymorphisms (SNPs) that can be used to differentiate between adapted and non-adapted B1K ecotypes.

## Results

### Physiological characterization of wild barley plants

**Figure 1:**
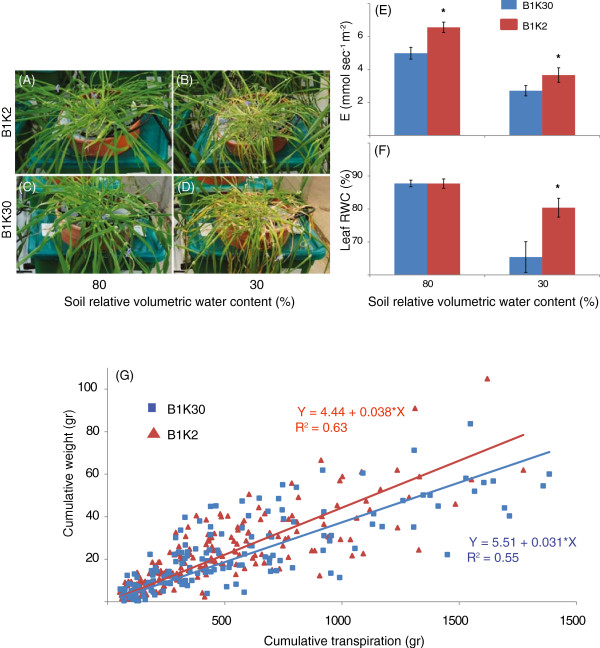
**Variation in transpiration, leaf RWC and WUE among wild barley ecotypes at different soil water contents. (A-D)** Pictures of ecotypes B1K2 **(A and B)** and B1K30 **(C and D)** under well-irrigated condition or full field capacity - 80% SWC **(A and C)** and drought condition - 30% SWC **(B and D)**. **(E)** Average mid-day whole-plant transpiration of B1K2 and B1K30 under normal (80% soil moisture content) and drought (30% soil moisture content) conditions. **(F)** Average mid-day leaf RWC of B1K2 and B1K30 under normal and drought conditions. **(G)** The WUE of each ecotype as determined by fitting a linear curve for the ratio between the plants’ cumulative weight gain and cumulative transpiration. The error bar and asterisk in **(E)** and **(F)** represent standard error (±SE) and significance differences (*t* test, p <0.05) among the ecotypes, respectively. Data **(E and F)** are means of four independent repetitions.

The B1K2 and the B1K30 accessions from the Barley1K collection [9] were selected based on previous studies for their difference in physiological responses to drought. The physiological measurements were conducted in greenhouses and plants were kept under natural light conditions and semi-controlled temperature and humidity (Figure 1A-D, see Methods). Measured traits included mid-day whole-plant transpiration (E), weight gain and loss, which were normalized to the plant leaf area, water-use efficiency (WUE), and leaf relative content (RWC; see Methods). Unexpectedly, the desert ecotype lost more water than the Mediterranean ecotype cultivar under both well-irrigated (80% volumetric water content) and drought conditions (30% volumetric water content; Figure 1E). Despite its higher transpiration rate, the desert cultivar maintained a higher RWC than the Mediterranean ecotype under drought conditions (Figure 1F). Moreover, the ratio between the cumulative weight gain and cumulative transpiration of plants (Figure 1G) revealed that the desert ecotype has a higher WUE than the Mediterranean ecotype with WUE calculated as the ratio of RWC to transpiration rate, E (One-way ANOVA, p =0.02). To identify candidate genes reflecting the differential response to drought stress, leaf samples from two plants of each ecotype were sampled for RNA extraction at day 5 of the drought treatment (Methods).

### 454 sequencing and *de novo*assembly of the wild barley transcriptome

**Figure 2:**
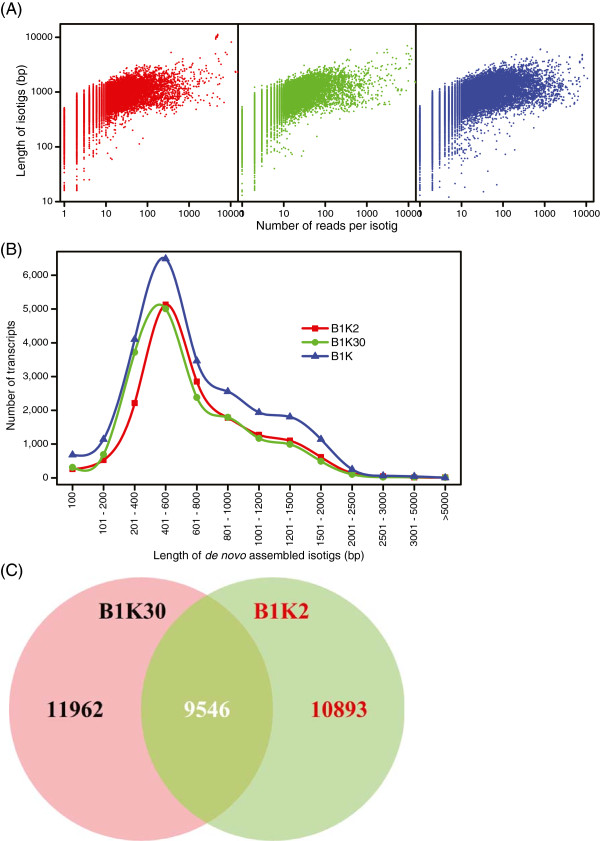
**Summary of assembled transcripts and homology among ecotypes. (A)** Log-log plot comparing isotig length and numbers of reads per isotig. **(B)** Frequency distribution of isotig lengths. **(C)** A Venn diagram showing homology among ecotypes.

Two cDNA libraries of the drought-stressed wild barley accessions were sequenced with 454 sequencing technology. A summary of the raw sequence data is presented in Table 1. Counts of raw reads and of the resulting unique transcripts were about the same in both libraries. After quality trimming, 99% of the reads had phred-like quality scores >20 and an average read length of 356 bp (B1K2), 313 bp (B1K30) and 338 (B1K). Almost 95% of both B1K2 and B1K30 were included in separate and joint *de novo* assemblies (Table 1). After clustering, we obtained three *de novo* assemblies with 20,439 putative unique transcripts (PUTs) for B1K2, 21,494 for B1K30 and 28,720 for B1K (joint assembly) that included isotigs and true singletons. Length distributions of the isotigs or PUTs in all three assemblies are given in Table 1, which shows that a joint assembly of the B1K2 and B1K30 libraries into a single assembly did not significantly increase average isotig length. There is also a strong positive correlation between the number of reads per isotig and the length of the isotig (Figure 2A), which affects the quality of subsequent functional and evolutionary analyses as presented below.

**Table 1:** **Summary of wild barley 454 transcriptome sequencing,** ***de novo*** **assembly and annotation**

Analysis	B1K2	B1K30	B1K
Reads:			
No. of raw reads (Mbp)	575,918 (223.40)	562,862 (193.91)	1,138,780 (417.31)
No. of reads after trimming (Mbp)	559,019 (199.22)	549,791 (171.81)	1,097,384 (370.75)
Mean trimmed reads length (bp)	356	313	338
Range of trimmed reads (bp)	20 – 1,080	20 – 1,009	20 – 1,080
No. of aligned reads (Mbp)	529,779 (191.06)	522,279 (164.34)	1,054,990 (356.35)
***De novo*** **assembly:**			
^1^No. of assembled reads (%)	529,297 (95)	522,093 (95)	1,035,947 (94)
^2^No. of isotigs (Mbp)	15,956	16,711	23,675
N50 (bp)	915	914	910
No of singletons	6,235	6,618	9,383
**Unigenes (≥100 bp):**			
^3^All	20,503	21,553	28,828
^4^Clustered	20,439	21,494	28,720
Mean size (bp)	619	562	609
Size (Mbp)	12.658	12.090	17.491
≥ 500 bp (%)	9,732 (63)	8,633 (54)	12,785 (59)
≥ 1000 bp (%)	3,044 (20)	2,621 (16)	4,609 (21)
**Annotation:** *number (%) of annotated transcripts*			
InterPro	4,300 (21)	5,137 (24)	7,153 (25)
Pfam	3,829 (19)	4,469 (21)	6,335 (22)
NR	13,637 (67)	13,532 (63)	^5^na
Swiss-Prot	8,143 (40)	7,906 (37)	na
GO	7,293 (36)	7,069 (33)	na
KGG	6,612 (32)	6,102 (28)	na

^1^assembled reads: fully and partially assembled; ^2^isotigs: groups of contigs and large contigs; ^3^all: clustered isotigs + singletons; and ^4^clustered: clustered isotigs + true-singletons (putative unique transcripts, PUTs). ^5^na: analysis not performed.

### Reference-based mapping of 454 sequences and SNP identification

In addition to the *de novo* assembly, we also employed a reference-based mapping approach. With this method, 94% of the B1K2 reads could be mapped against the B1K30 unique transcripts (Table 2). Over 75% of the reads were mapped onto the cultivated barley full length cDNA (Hv. fl-cDNA) sequences and about 65% against the coding sequences (CDS) of the high-confidence (HC) cultivated barley genes. A substantial proportion of reads mapped to more than one target (15 to 47%; Table 2). Nearly all reads (98%) mapped against the Morex whole-genome shotgun sequence, although the number of reads mapped at several locations was two-fold higher than reads mapped only once.

**Table 2:** **Summary of reference-based mapping of wild barley reads against cultivated barley sequences**

Reference →	B1K30	Hv. fl-cDNA			Barley HC genes			Chr. Hv. WGS		
Reads →	B1K2	B1K2	B1K30	B1K	B1K2	B1K30	B1K	B1K2	B1K30	B1K
^**1**^ **Aligned reads (%):**										
All	93.6	77.5	76.6	77	67.6	63	65.3	80.0	79.4	79.7
1x	52.7	47.1	47.5	47.3	51.8	48.6	50.2	32.7	35.7	34.2
>1x	40.9	30.3	29.2	29.7	15.8	14.4	15.1	47.3	43.7	45.5
**SNPs:**										
Raw SNPs	28,289	27,279	24,637	40,639	16,284	14,509	24,446	39,545	36,021	61,852
Filtered SNPs	1,017	1,067	1,717	9,092	1,184	1,081	5,036	4,053	4,372	15,330
^2^Gene					132	299	1,805	1,010	1,488	4,389
SNPs/gene					9	3.6	2.8	4.0	2.9	3.5
SNPs/kb	4.4	3.5	2	1.78	7.3	2.8	2	3.5	2.6	2.4

^1^Aligned reads (%): proportion of all aligned reads, reads aligned 1x and over 1x.

^2^Gene: number of genes with SNPs.

Mapping against chromosomal barley genome (Hordeum vulgare 030312 v2.16) was used for SNP effect annotation.

### Identification of wild barley-specific transcripts

One goal of this study was to identify transcripts that are unique to wild barley. All PUTs were compared to each other with BLAST to identify the proportion of homologous PUTs shared among the two divergent wild barley ecotypes. The two divergent ecotypes shared about 9,546 (29%) of the PUTs from both ecotypes or 46% of B1K2 PUTs (Figure 2C). Hence, the majority of the transcripts were restricted to one of the two ecotypes. The mean length of shared transcripts among ecotypes (B1K2 = 800 bp and B1K30 = 741 bp) was significantly larger (p <0.001) than ecotype-specific transcripts (B1K2 = 461 bp and B1K30 = 420 bp). The proportion of shared PUTs increased to 69 and 84% for transcripts longer than 500 and 1,000 bp, respectively. This suggests either that ecotype-specific transcripts are shorter or that the power to identify orthologs in the other ecotype depends on transcript length.

We further compared the wild barley transcripts with cultivated barley genes using a reciprocal BLAST hit (RBH) approach to identify putative orthologs (Table 3). A total of 82% (16,781) of B1K2 and 81% (17,318) of B1K30 transcripts were orthologous to at least one of three cultivated barley sequence datasets. A proportion of 16% (3,245) of B1K2 and 17% (3,674) of B1K30 transcripts did not have a significant RBH in the other wild barley ecotype and the cultivated barley data. They can be considered as candidate ecotype-specific genes. Only a small proportion of 2% of transcripts matched the other ecotype but not cultivated barley. Among the combined B1K PUTs, 25% (7,102) did not match to cultivated barley. These results suggest that B1K2 and B1K30 are highly divergent ecotypes and that a significant proportion of wild barley transcripts are not homologous to currently known genes from the cultivated barley genome. Since this proportion may be influenced by variation in transcript length due to incompletely sequenced cDNAs (i.e., experimental error), we asked whether transcript length is correlated with the proportion of reciprocal BLAST hits to cultivated barley using PUTs from the joint assembly (B1K). The proportion of orthologous hits to cultivated barley increased with sequence length from 41.3% for sequences of 100–250 bp length to 82.5% with sequences ≥1,000 bp length (Additional file 1: Figure S2). The mean length of ecotype-specific PUTs was significantly shorter than of those occurring in both ecotypes (p <0.0001). The same pattern was observed among joint assembly PUTs with (695 bp average length) and without (347 bp) a hit to cultivated barley (p <0.0001). Therefore, sequence length variation needs to be considered in the identification of transcripts specific to wild barley. On the other hand, 73 out of 7,102 (1%) long wild barley (B1K) transcripts (≥1000 bp) currently have no orthologs in cultivated barley sequence data suggesting they are good candidates wild barley-specific genes.

**Table 3:** **Homology within and among wild and domesticated barley sequences**

Source	B1K2	B1K30	Hv*	No. of PUTs (%)	Transcript type
B1K2	√	x	x	3,245 (16)	B1K2-specific
	√	√	x	413 (2)	wild barley-specific
	√	x	√	7,648 (37)	B1K2- & Hv.-specific
	√	√	√	9,133 (45)	in both wild & cultivated barley
B1K30	x	√	x	3,674 (17)	B1K30-specific
	√	√	x	502 (2)	wild barley-specific
	x	√	√	8,288 (39)	B1K30- & Hv.-specific
	√	√	√	9,030 (42)	in both wild & cultivated barley
B1K	√		x	7,102 (25)	wild barley-specific
	√		√	21,617 (75)	in both wild & cultivated barley

*Hv: RBH against sequence data from barley HC, fl-cDNA and HarvEST v1.83 assembly 36.

The orthologous analysis was based on RBH.

### Conservation of unique transcripts in evolutionary distant plant species

To estimate how many wild barley transcripts are conserved in more distant plant species, we compared them against five fully sequenced and annotated plant genomes of variable evolutionary distance to barley: *Brachypodium*, rice, sorghum, maize and *Arabidopsis*. Based on the RBH approach with an e-value ≤1e-6, ≥75% identity and alignment length of ≥33 amino acids as cutoffs, the highest proportion of orthologous genes was observed between wild barley and *Brachypodium*, followed by rice, sorghum, maize and *Arabidopsis*, in decreasing order (Additional file 2: Table S1), which reflects the evolutionary distance of these species to barley. A total of 13% (19) long (≥1 kb) unique transcripts from the B1K assembly for which no ortholog was found in barley HC genes and fl-cDNA were homologous to other grass species. We also investigated which proportion of ecotype- and wild barley-specific transcripts are conserved across fully sequenced and annotated grass genomes. Based on RBH, 98% each of ecotype-specific (B1K2 = 3,191 and B1K30 = 3,606) and wild barley-specific (B1K =6,993) transcripts have no orthologs in *Brachypodium*, rice and sorghum. The results indicate that a significant proportion of putative unique ecotype- and wild barley-specific transcripts are not conserved in closely related grass species (Additional file 3: Table S2).

### Annotation with GO terms and KEGG pathways

All wild barley transcripts were annotated based on homology searches in the non-redundant NCBI protein (NR), Swiss-Prot/Uniprot, KEGG and InterPro/Pfam databases using BLASTX. The results are summarized in Table 1 and Additional file 1: Figure S3A. Proportions of significant hits ranged from 40% (Swiss-Prot) to 67% (NR) for B1K2 PUTs, and from 37% (Swiss-Prot) to 63% (NR) for B1K30 PUTs. The majority of unique transcripts (87% of B1K2 and 88% of B1K30) longer than 500 bp showed significant BLASTX hits in the NR database, whereas the majority of PUTs without BLASTX hits in the NR database (81.6% of B1K2 and 87.2% of B1K30) were shorter than 500 bp (Additional file 1: Figure S3B). Therefore, transcript length affects the functional annotation by homology search because shorter transcripts may have a lower chance of a BLAST hit or are incomplete cDNAs without a protein-coding region.

We used BLAST2GO to assign Gene Ontology (GO) terms to protein-coding unique transcripts (Table 1 and Additional file 1: Figure S4). In both ecotypes, the majority of GO terms describe molecular function (36% of unique transcripts), followed by cellular component (34%) and biological process (29%). Among molecular function terms, the majority of transcripts was annotated with 'binding’ (GO: 0005488; 46%) and 'catalytic activity’ (GO: 0003824; 43%). Among biological process terms, most transcripts were annotated with the terms 'metabolic process’ (GO: 0008152; 41%), 'cellular process’ (GO: 0009987; 39%) and 'response to stimulus’ (GO: 0050896). The proportion of genes annotated with these terms was essentially identical for the B1K2 and B1K30 transcripts.

### Identification of stress-related genes and transcription factors

To identify unique transcripts that are orthologous to previously annotated stress-related genes and transcription factors (TFs), we used a stringent BLASTX search measuring parameters combined with a RBH approach. Based on the list of candidate genes described in Materials and Methods, we found 43 stress-associated transcripts both in B1K2 and B1K30 unique transcripts, of which seven were found in B1K2, 18 only in B1K30 and 18 in both ecotypes (Additional file 3: Table S3). The majority of unique transcripts were homologous to heat shock proteins followed by aquaporin and *ERD* genes from different grasses; *LEA* and *ABC* transporter genes were also detected. To further identify wild barley putative transcripts involved in stress response, the literature was searched for functional genes, transcription factors and enzymes that regulate abiotic stress tolerance in different plants. Wild barley transcripts orthologous to these identified stress-responsive genes, transcription factors and enzymes were identified in the GO, KEGG and protein domain (Pfam) annotation results. We obtained 839 B1K2 and 881 B1K30 unique transcripts associated with more than 30 different stress-responsive genes (Additional file 2: Table S4). Most transcripts matched to zinc-finger domain containing genes (185 in B1K2 and 241 in B1K30), ABC transporters (104 and 93), and heat shock proteins (61 and 63). Likewise, 165 transcripts in B1K2 and 156 in B1K30 could be assigned to 14 different transcription factors responsible for abiotic stress tolerance.

Since response of drought tolerance is regulated by TFs, we used the RBH approach to find orthologous of known barley TFs among wild barley PUTs. We identified 203 (out of 780) known barley TFs orthologous to 165 B1K2 and 155 B1K30 unique transcripts and 312 TFs from *Arabidopsis thaliana* and five grasses (*Brachypodium*, rice, sorghum, maize and wheat) that were homologous to 165 B1K2 and 170 B1K30 unique transcripts (Additional file 3: Table S3).

### Gene prediction based on barley HC genes

**Figure 3:**
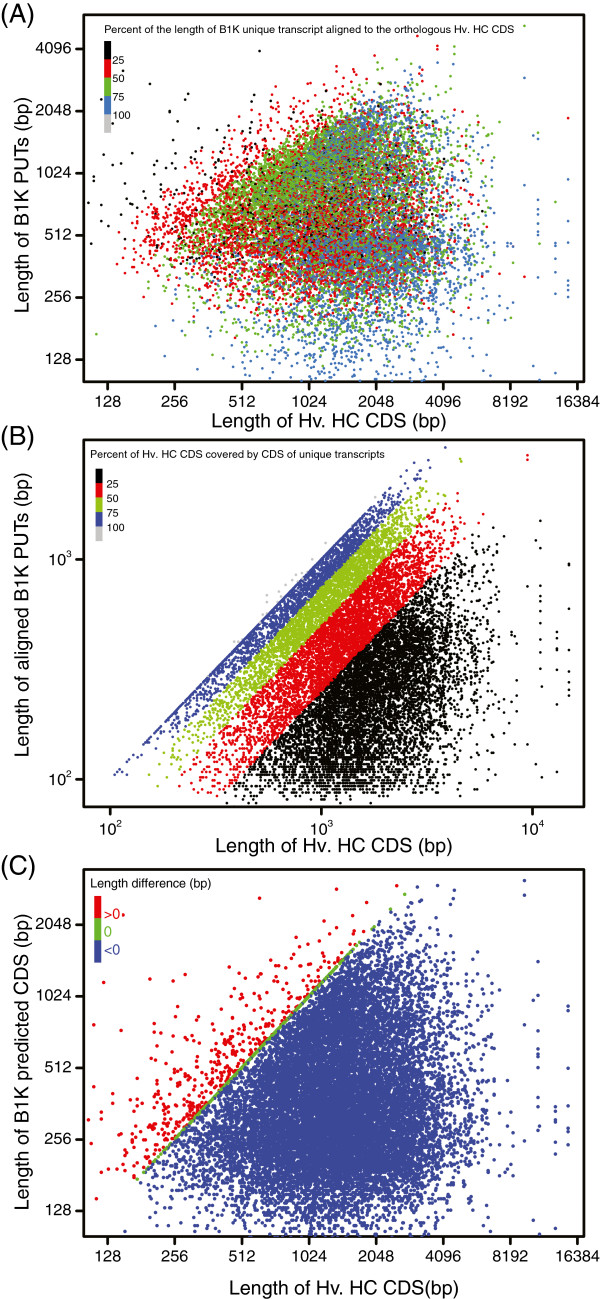
**Log-log plot of correlation among B1K PUTs and barley genes. (A)** Correlation among the length of B1K PUTs and Hv. HC CDS. **(B)** Correlation among the length of B1K PUTs CDS predicted based on BLASTX and Hv. HC CDS. **(C)** Correlation among the length of B1K PUTs CDS predicted by OrfPredictor and Hv. HC CDS. The color bars showed the proportion of B1K PUTs length aligned to the orthologous HC CDS **(A)**, the proportion of HC CDS covered by aligned PUTs **(B)**, and the length difference (bp) among CDS of PUTs and HC genes.

To evaluate the use of wild barley transcriptome sequences for improving the cultivated barley genome annotation, we first investigated how many barley HC genes were fully and partially covered by our unique transcripts. We achieved this by predicting the extent of coding sequence (CDS) covered by the unique transcripts using BLASTX (Additional file 2: Table S5). 62% of B1K PUTs tagged 45% of barley HC genes, and 5% of B1K PUTs homologous to barley HC genes fully covered HC genes. The majority of shorter HC genes (i.e., less than 1500 bp) were well covered by PUTs while the longer HC genes were less covered (Figure 3A and 3B). A higher proportion of longer than shorter HC genes has a hit with wild barley PUTs (Figure 3B).

In addition, we used OrfPredictor to predict CDS (Additional file 2: Table S5). Over 99% of the unique transcripts from all three assemblies consist CDS longer than 33 nucleotides with an average predicted CDS length of 350 bp (B1K); 19% were longer than 500 bp and 4% were longer than 1 kb. 3.3% (584) of the predicted CDS from B1K PUTs had the same CDS length as their orthologous HC CDS, while 2.6% (456) were longer than their orthologs (Figure 3C and Additional file 2: Table S5). We also analysed the proportion of novel transcripts annotated with CDS. Almost all (98%) of them were annotated with CDS ≥33 bp and 85% with CDS ≥200 bp (Additional file 2: Table S5). The results indicate that *ab initio* prediction of CDS identifies a substantial number of transcripts that differ from annotated orthologous barley genes and may contribute to an improved annotation (Additional file 1: Figure S5A-D).

### SNP discovery in transcriptome data

**Figure 4:**
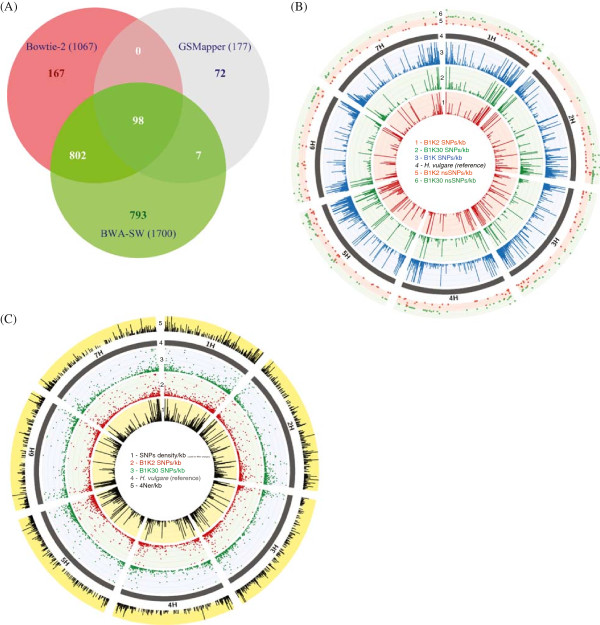
**SNPs identified from the two wild barley ecotypes. (A)** A Venn diagram showing the number of SNPs identified from assembly made by three different reads mapping programs and their combinations. SNPs are identified after stringent filtering (sequence depth ≥8x and a minimum of four reads supporting the reference and variant SNPs) and SNPs in the intersection of two or more ellipse belong to the SNPs called from mapping made by two or more programs. **(B)** Circos diagram showing the frequency of stringently filtered SNPs per kb (depth ≥8x from which minimum of four reads each supporting reference and variant SNPs). **(C)** Circos diagram showing the frequency of wild barley SNPs density per kb, filtered SNPs per kb (depth ≥8x from which minimum of two reads each supporting reference and variant SNPs) and estimated recombination rates (4*N*_*e*_ *r*) per kb. Histograms are showing SNP frequencies among wild and cultivated barley. Recombination rates are estimated among wild barley. For the display, the maximum SNP frequencies are set at 20 (B, SNPs/kb), 10 (B, nsSNPs/kb), 30 (C, SNPs/kb), 20 (C, SNPs density/kb) and 4 (C, 4*N*_*e*_ *r*/kb) – these maximum values are higher than the 3rd quartiles of the respective data.

We searched for SNPs in the transcriptome sequences to differentiate the two wild barley ecotypes. As a first step, we compared three SNP calling tools (Bowtie-2, BWA-SW, and GSMapper, see Methods) by analyzing B1K2 reads aligned onto Hv. fl-cDNA. After a very stringent filtering, a total of 1,939 SNPs were identified, of which 53% (1,032 SNPs) were unique to a single tool, while 47% (907 SNPs) were identified by more than one tool (Figure 4A). SNP numbers differed ten-fold between tools, and only 5.1% (98 SNPs) were identified by all three tools (Figure 4A). Under the assumption that SNPs identified by at least two tools are likely true SNPs, Bowtie-2 appears to be the most accurate SNP caller. We identified 1,017 high quality SNPs from 28,289 raw SNPs between the two wild barley ecotypes (B1K2 and B1K30) with Bowtie-2 and stringent SNP filtering criteria. This corresponds to a frequency of 4.4 SNPs per 1 kb (Table 2). SNP counts identified by using the cultivated barley sequences as reference are shown in Table 2. The B1K set produced a proportionally larger number of filtered SNPs than the B1K2 and B1K30 libraries alone, which reflect the sum of both accessions as well as the higher coverage per nucleotide position in the combined dataset (Figure 4B, Additional file 1: Figure S6 and Additional file 1: Figure S7). We also compared how SNPs identified from desert B1K2 and Mediterranean B1K30 ecotypes overlap with SNPs discovered from wild barley ecotype B1K4 , which belongs to the same Barley1K collection and was shotgun sequenced at low coverage [23]. A quarter of SNPs identified in each ecotype (9,775 in B1K2 and 8,682 in B1K30) overlapped with SNPs identified in B1K4 (Additional file 2: Table S6) using a different sequencing approach. This overlap suggests that a significant proportion of SNPs in the transcriptome sequence data are correctly inferred.

### SNP annotation

In the B1K set, 15,330 SNPs were identified from 61,852 raw SNPs after filtering in the B1K set. The top three SNP categories were SNPs in 3′ untranslated regions (3′-UTR; 48%), synonymous SNP (sSNP; 29%), and non-synonymous SNP (nsSNP, 14%). Other (9%) type of SNPs included intronic and intergenic SNPs, possibly because of unprocessed mRNAs in the libraries (Table 4). The high proportion of 3′-UTR SNPs likely resulted from abias towards the 3′-end of transcripts due to the cDNA library construction method [28]. In both ecotypes, the proportion of sSNP in genic and CDS/exon regions was over 2-fold higher than the proportion of nsSNP (Table 4). The average number of nsSNP in the genic regions was by 2-fold higher in the desert ecotype B1K2 than in the Mediterranean ecotype B1K30. Similarly, other SNP types with strong functional effects (start codon loss, stop codon gain, splice site acceptor and splice site donor) were 2-fold more frequent in B1K2 than in B1K30 (Wilcoxon test, p = 0.004). The average proportion of nsSNP/sSNP ratios was higher in stress-related genes of both ecotypes (B1K2 = 0.38:0.62 and B1K3 = 0.45:0.55) than the average proportion across all genes (0.32:0.68 in both ecotypes). Some of these genes belong to highly conserved protein families such as heat shock/chaperone, aquaporin, bZIP, Myb, Chlorophyll a-b binding and photosystem II (PSII) chlorophyll apoproteins suggesting that these gene classes be involved in adaptive evolution. The chromosomal distribution of nsSNPs is shown in Figure 4B, the distribution and proportion of sSNP and nsSNP in selected barley genes is further summarized in Additional file 1: Figure S8, and 30 barley genes with the largest numbers of putatively function-affecting SNPs are summarized in Additional file 2: Table S7.

**Table 4:** **Summary of SNP effects in wild barley 454 transcripts**

SNP effect	B1K2	B1K30	B1K	B1K2	B1K30	B1K	B1K2	B1K30	B1K
	In all regions (#)			In genic region (%)			Mean per genic region		
Synonymous	1,514	1,225	3,943	45.49	32.63	28.81	2.411	1.284	1.298
Non-synonymous	699	564	1,919	21.00	15.02	14.02	1.113	0.591	0.632
3′ UTR	816	1,546	6,570	24.52	41.18	48.01	1.299	1.621	2.163
5′ UTR	154	149	458	4.63	3.97	3.35	0.245	0.156	0.151
Non-synonymous start	1	2	2	0.03	0.05	0.01	0.002	0.002	0.001
Start lost	0	1	1	0.00	0.03	0.01		0.001	0.000
Stop gained	8	14	27	0.24	0.37	0.20	0.013	0.015	0.009
^1^Splice site acceptor	2	3	10	0.06	0.08	0.07	0.003	0.003	0.003
^2^Splice site donor	3	6	13	0.09	0.16	0.09	0.005	0.006	0.004
Start gained	26	31	95	0.78	0.83	0.69	0.041	0.032	0.031
Intron	105	213	648	3.16	5.67	4.73			
^3^Upstream	753	1,041	3,445				0.746	0.70	0.785
^4^Downstream	1,504	1,942	7,381				1.489	1.31	1.682
Intergenic	1,173	1,272	3,854				0.167	0.223	0.213
**Summary – SNPs with/in:**									
^5^High/moderate impact	712	588	1,970				1.134	0.616	0.648
^6^Gene region	5,585	6,737	24,512						
^7^Genic region	3,328	3,754	13,686						
^8^CDS/exon regions	3,192	3,501	12,920						
^9^Gene with genic SNPs	628	954	3,039						

^1^Splice site acceptor: variant hits a splice acceptor site (two bases before exon start, except for the first exon).

^2^Splice site donor: variant hits a Splice donor site (two bases after coding exon end, except for the last exon).

^3^Upstream: variant hits Upstream of a gene (within length of 5Kb).

^4^Downstream: variant hits Downstream of a gene (within length of 5Kb).

^5^High/Moderate SNPs effect: nsSNP CDS + Spice acceptor + splice donor + start lost + stop gained.

^6^Gene region: Upstream +5 UTR + Exon + Intron +3 UTR + Downstream.

^7^Genic region: 5 UTR + Exon + Intron +3 UTR.

^8^CDS/exon region: 5 UTR + Exon +3 UTR.

^9^Number of genes with SNPs affecting the UTRs, exon and intron.

SNP effects are based on mapping of 454 NR reads against chromosomal barley genome (Hv. 030312 v2.16).

### Estimation of the population recombination parameter

We estimated the population recombination parameter *ρ* =*4N*_*e*_*r* from three wild barley ecotypes (B1K2, B1K30, B1K4) using 7,440 SNPs, and found an average value of 0.089 ρ/kb ±1.02 (SD). It varies between chromosomes with the highest and lowest rates in chromosome 1 (0.169) and 5 (0.062), respectively. The recombination rates on the remaining chromosomes were 0.073 (2), 0.084 (3), 0.083 (4), 0.082 (6) and 0.067 (7). Nonetheless, the recombination rate per kb does not differ between chromosomes (one-way ANOVA, p =0.178). High recombination rates were observed in telomeric regions of each chromosome (Figure 4C). There is no significant correlation (Pearson’s *r* = -0.03, p =0.12) between SNP density and the population recombination parameter (Additional file 1: Figure S9).

## Discussion

### Phenotypic response to drought stress in wild barley

Wild barley ecotypes from the divergent Mediterranean and desert climates are phenotypically different in several quantitative traits [18, 20]. Recent work has corroborated this finding by genomic and phenotypic analyses [8, 9, 20, 29]. Furthermore, drought stress tolerance in barley likely appears to be linked to WUE because wild barley genotypes from contrasting environments and with different WUE differed in their level of drought stress tolerance [30]. This study led to the discovery of the barley dehydration-responsive *Hsdr4* gene, which is more strongly expressed in drought-tolerant than sensitive genotypes. The physiological responses in the present study are consistent with these results and contribute to the list of ecologically relevant phenotypic differences between Mediterranean and desert barley that may reflect differential adaptation. Although the desert ecotype showed a higher absolute water loss, its WUE and RWC were significantly larger under drought conditions. In contrast to our results, Eppel *et al.* [29] observed a similar decrease of WUE under drought stress in both Mediterranean and desert wild barley ecotypes, whereas other physiological traits such as Photosystem II yield and non-photochemical quenching (NPQ) differed significantly between both ecotypes. Currently, no studies are available that investigated the relationship between plant fitness in dry environments and WUE or RWC in wild barley. WUE was positively correlated with differential fitness in *Boechera stricta* [31], suggesting a contribution to fitness, but this was not observed in the desert annual *Helianthus anomalus* [32] *.* Taken together, these contrasting results highlight the complex nature of the trait. Future studies will have to investigate the genetic relationship between fitness and physiological traits in differentially adapted wild barley using, for example, segregating populations derived from crosses between differentially adapted parental lines or accessions.

### Sequencing and assembly of wild barley transcriptome

The sequencing of normalized cDNA libraries resulted in more than half million reads per library of which almost 95% of high-quality trimmed reads could be *de novo* assembled into putative unique contigs. This high proportion of assembled reads is similar to other studies utilizing 454 transcriptome sequencing in species with complex genomes such as Douglas-fir [33], and significantly higher than in other studies [34, 35]. The unique transcripts length increased with the number of assembled reads per transcripts, in agreement with a previous study [33]. The present study with an average contig length of 609 bp and a N50 value of 910 bp in the B1K assembly is significantly larger than in a previous 454 transcriptome analysis of cultivated barley with a mean contig size of 505 bp and N50 values of 531 bp [36], or in a similar survey in two grass species of the genus *Spartina* [34]. The numbers of assembled unique transcripts in each ecotype are quite close to the 26,159 barley HC genes, and the number of putative unique transcripts from the combined B1K assembly is even higher. The full-length transcript data from cultivated barley (Hv. fl-cDNA) originate from complete genome sequencing efforts [37, 38] that involved several sequencing runs using different sequencing approaches and were therefore not comparable with transcripts obtained in a single 454 run. In the present study the libraries were made from leaf tissue only. We therefore expect that a significant proportion of barley genes may not have been captured, since only 72-84% of barley HC genes were expressed in more than one developmental stage or tissue [23].

Because no reference genome for *H. spontaneum* was available, we used the Hv. fl-cDNA, Hv. HC CDS and the currently released barley genome as references for read mapping and SNP discovery. When mapped against the full-length cDNA, over three-fourth (76%) of both B1K2 and B1K30 non-redundant reads were mapped; whereas only around two-third were mapped against Hv. HC genes, and about 80% onto chromosomal genomic sequences. Within wild barley, the majority (93%) of B1K2 non-redundant reads were mapped onto the B1K30 transcriptome from *de novo* assembly. A substantial proportion of reads were mapped to many sites of the reference sequences: 41% reads in mapping among ecotypes and up to 47% reads in mapping of ecotype onto WGS. Similar results have been reported for chickpea (47-60%) [39] and maize (52%) [40]. The presence of paralogous genes and isoforms from splice variants, and quality of the reference sequences could contribute to the observed duplicate mapping [39–41] and some 454 reads mapping to multiple sites may represent transcribed transposable elements.

### Identification of new barley genes by transcriptome sequencing

Since the cDNA libraries were normalized, a significant proportion of rare transcripts should have been sequenced with higher probability than non-normalized libraries and contributed to the discovery of novel transcripts and genetic variants [28, 42]. The homology searches against all three current barley databases, barley HC genes, Hv. fl-cDNA and HarvEST, supported this expectation because a substantial proportion of the unique transcripts (25% or 7,102 in the B1K set) has not been annotated as genes in cultivated barley, although 98% of the reads were mapped onto the cultivated barley genome. Furthermore, the majority of new transcripts from both ecotypes did not show significant BLAST hits in closely related grass species (98% without homology in *Brachypodium*, rice and sorghum) and protein databases (87% without homology in NR, Swiss-Prot and InterPro). On the other hand, 85% of new or novel transcripts were annotated with a coding sequence length longer than 100 bp. Although these results look unexpected, similar results were reported in cultivated barley [36] with 40% new contigs, as well as in wheat [35], the grass *Spartina* [43] and the zebra finch [42]. Since all of these studies employed 454 sequencing of normalized cDNA libraries, it appears to be an efficient approach for the discovery of new genes. The total number of barley genes was estimated to be around 30,400, and 26,159 (86%) of them are reported as high-confidence (HC) genes [23]. The discrepancy between the high proportion of reads mapped to the *H. vulgare* genome and the large number of novel transcripts specific to wild barley found in our data may be explained as follows: (i) The novel transcripts represent genes that are present in wild but not in cultivated barley, or (ii) are present but have not yet been identified in cultivated barley because the 'Morex’ WGS data, to which 98% the raw 454 reads could be mapped, still represent a draft genome assembly; (iii) the novel transcripts may be derived from genes with structural variants and alternative splicing. The local alignment approach used to map 98% of the reads against the WGS data 'soft-trimmed’ the read end, which leads to a high proportion of aligned reads by removing non-matching ends. It has been shown before that such trimmed reads are associated with structural variants and alternative splicing, both of which are highly prevalent in barley [23, 25]; (iv) Novel transcripts without any homology may represent poorly conserved non-coding RNAs (the 'dark matter’ of genome) [44] or untranslated regions (UTRs) of the genome because they originated from incompletely transcribed or sequenced mRNAs and may be too short to generate significant BLAST hits.

Based on the RBH analysis, our assemblies represent 41-47% orthologs of the current set of 26,159 barley HC genes. A comparison of our GO annotation with the barley Affymetrix GO terms [45] showed a similar distribution suggesting that our transcripts well represent wild barley genes. Known barley genes not present in the transcriptome data may have been lost or not sampled during library preparation or sequencing. Alternatively, they may not be expressed in wild barley leaf under drought stress, or may show low expression or expression at different developmental stages or plant tissues, since only 72-84% of the HC genes were expressed in all developmental stages or tissue samples [23].

The unique transcripts generated from the differentially adapted wild barley ecotypes contribute to an improved annotation of the barley genome. This is because many PUTs identified in this study are not orthologous to barley HC genes and/or fl-cDNA, but some of these non-orthologous transcripts are conserved in grasses and other plant species. A significant number of CDS in our transcript set are longer than their orthologous barley HC genes, indicating that some of the annotated barley genes are not complete or differentially spliced.

A substantial proportion of PUTs are not shared between the two wild barley ecotypes. They may (i) represent genes whose transcripts were lost during cDNA normalization or library preparation, (ii) presence/absence polymorphisms, i.e., ecotype-specific or non-shared transcripts that reflect genome divergence due to differential loss or gain of transcripts, as has been documented in maize [46–48], or (iii) differential expression of genes in both accessions in response to the drought treatment. Since we sequenced normalized cDNA libraries and did not include untreated plants, our data do not allow us to differentiate between presence/absence polymorphisms and differential expression. Genome re-sequencing using targeted exome capture and RNAseq of treated and untreated plants will reveal why transcripts are not shared between the two ecotypes.

### Identification of candidate stress-related genes and transcription factors

Studies in different model organisms such as rice and *Arabidopsis thaliana* uncovered numerous genes encoding transcription factors, signal transduction and transporter proteins with well characterized roles in drought stress regulation [49]. Transcriptome sequences generated from ecotypes adapted to different environments can be used to identify homologs of known stress-related genes in non-model organisms. Using RBH- and keywords-based searches, we identified more than 800 genes with known stress-related genes from other species in each accession. Genes encoding zinc-finger proteins, ABC transporters, heat shock proteins and transcription factors (e.g., MYB, MAPK, bHLH, bZIP, NAC, WRKY) were the most abundant types of stress-related genes. As an example, the ABC subfamily G (ABCG) transporter significantly contributes to leaf water retention in wild barley and rice [50]. Some of these transcription factors, for instance, bZIP, bHLH and MYB are found to be regulating different stress responses in plants [51, 52]. As discussed above, the presence of different homologs to known stress-related genes in the two ecotypes does not necessarily mean that they are involved in drought-response or indicative of the genetic divergence between the two ecotypes, but are putative candidates for further evolutionary or functional investigations. For instance, the barley dehydration-responsive *Hsdr4* gene, which encodes a Rho-GTPase-activating protein and is highly expressed in drought tolerant relative to sensitive genotypes under drought stress [30, 53] is also present in the PUTs identified in this study. In previous studies, SNPs were only found in the intron and promoter regions of tolerant and sensitive genotypes, but the comparison of PUTs and *Hsdr4* identified six new SNPs in the coding region.

### SNP discovery in transcriptome sequences

Despite the great power of current sequencing technologies, SNP identification is still challenging and affected by several factors [54]. We found ten-fold different numbers of SNPs between SNP callers indicating that SNP calling strongly depends the particular algorithm [33]. Early SNP calling methods such as GSMapper that use fixed cutoff rules tend to underestimate SNP numbers when compared to probabilistic methods as implemented in Bowtie [54]. Probabilistic methods also depend on parameter settings because numbers of SNPs identified with Bowtie 2 differed by 2.5-fold between local and end-to-end methods. One solution to this is the use of several tools because SNPs discovered by two or more programs are more likely true SNPs than those predicted by one only [39] and SNPs called at higher coverage most likely represent true SNPs [55].

We identified over one thousand SNPs in wild barley, resulting in 4.4 SNPs per kb at high coverage. This is similar to a recent survey of wild barley collected across Israel (one SNP per 239 bp, θ_*w*_ =0.00418) [10]. We also identified over one thousand SNPs by mapping against the cultivated barley sequences (Hv. fl-cDNA and Hv. HC genes). A quarter of SNPs from each ecotype are shared with SNPs identified from wild barley ecotype B1K4 used for barley genome sequencing [23], which independently confirms a significant proportion of discovered SNPs. Based on the comparisons of each ecotype against all three cultivated barley datasets, the SNP density of the desert ecotype B1K2 against cultivated barley is 1.9-fold higher than of the Mediterranean ecotype B1K30, which was calculated as the mean of the SNP densities of 1.8-fold to fl-cDNA, 2.6-fold to HC and 1.4-fold to the chromosomal genome sequences. There is more genetic variation in the desert barley and a higher genetic similarity between Mediterranean wild barley and cultivated barley suggesting that (i) the cultivated barley domestication occurred in the northern part of Israel, (ii) there is gene flow among cultivated and Mediterranean ecotypes, and/or (iii) the desert ecotypes diverged more because of the accumulation of adaptive and linked neutral variation through adaptive evolution to the desert conditions. Based on the proportion of shared SNPs, the B1K4 ecotype from Ein Prat appears more similar to the desert ecotype B1K2 than to B1K30.

These differences are supported by the analysis of SNP types. The higher absolute frequency of nsSNPs and of SNPs with functional effects, and the higher ratio of nsSNPs to sSNPs in the desert than Mediterranean ecotype reflects the larger genetic distance of the desert ecotype to cultivated barley. The higher number of functional polymorphisms in the desert ecotype may result from of a smaller effective population size causing a higher frequency of slightly deleterious amino acid polymorphisms due to reduced purifying selection, or a stronger and more frequent genome-wide positive selection as a consequence of adaptation to a stressful environment.

It should be noted that the observed distances are likely biased because of the SNP calling problems, the small sample size and the sequencing of a subset of genes captured by the stress-induced library. On the other hand, the larger genetic distance between the desert ecotype to cultivated barley than between the Mediterranean ecotype to cultivated barley is consistent with the phenotypic differentiation of the desert and Mediterranean wild barley types to cultivated barley for several quantitative traits [20]. SNP density at telomeric chromosomal regions is higher than in other regions in agreement with genome sequencing surveys of both cultivated and wild barley [23]. It may result from a higher gene density [23] or increased recombination rates in telomeric regions (Figure 4C). SNP density within wild barley is 2.2-fold greater than among wild (B1K) and cultivated barley. However, it is similar to the SNP density predicted in cultivated barley from the assembly of public barley EST sequences, one SNP per 240 bp (i.e., 4.1 SNPs/kb) [56]. This estimate may be biased by using public EST sequences originating from several genotypes for SNP identification.

## Conclusion

Physiological analysis of drought stressed desert and Mediterranean wild barley ecotypes suggested the existence of genomic differences and the resulting 454 transcriptome sequencing led to the discovery of novel transcripts in both ecotypes. The desert ecotype has a relatively higher SNP density and more SNPs with significant effects on protein coding genes than the Mediterranean ecotype. Based on functional and evolutionary conservation, several stress-related candidate transcripts and transcription factors were identified. The data generated in this study are valuable genomic resources for further improvement of the barley transcriptome and genome annotation. Using this resource, exome capture arrays can be designed to investigate presence/absence polymorphisms of putative wild-barley and ecotype-specific genes as well as to analyze genetic diversity in different groups of genes. The markers generated in the putatively stress-related genes can be used for the genetic analysis of drought adaptation and provide an avenue for the introgression of useful genetic variation into cultivated barley breeding populations.

## Methods

### Plant material

The two wild barley ecotypes used for this study were single seed descendents from the original Barley1K (B1K) wild barley collection [9]. Seeds from the desert ecotype (B1K2) were collected from Yerucham in the Negev desert (34.865265 E, 30.933476 N) and the Mediterranean ecotype (B1K30) from Nahal Oren ('Evolution Canyon’) near Haifa in Northern Israel (34.975673 E, 32.715772 N). The mean annual rainfall at B1K2 and B1K30 locations are 112 and 623 mm, while the water content in the soil are 0.543% and 10.379%, respectively [9].

### Physiological measurements

Sowing and cultivation of plants followed a previously described protocol [20] with few modifications. Seeds were planted in soil in planting trays and kept in the dark for 10 days at 4°C before they were incubated in a phytotron for 21 days at 16°C/10°C and short day conditions (9 h light; thereafter, seedlings were transplanted to 4 liter pots, which were irrigated twice per day in long day conditions (16 h light) at 22°C/16°C. The pots were filled with a commercial potting soil (Matza Gan; Shaham, Givat-Ada, Israel) and each pot contained one plant. The physiological measurement was conducted in greenhouses located at the Faculty of Agriculture, Food and Environment in Rehovot, Israel during December 2012 and January 2013. In the greenhouses, plants were kept under natural light conditions and vents and/or cooled moist air were used to ensure that the maximum temperature in the greenhouse did not exceed 35°C. The temperature and relative humidity were in the range of 18-35°C and 20-35% during the experiment.

After the plants were kept in the greenhouse for two weeks and when the nodes started to appear, the phenotypic measurements were started and further conducted for two months. Mid-day whole-plant transpiration (E) was calculated based on the difference between two weight readings, 90 min apart, taken between 11:00 and 13:00. The weight loss of each plant was normalized to the plant leaf area, which was taken using a leaf scanner (LI-COR 3100 Area Meter, LI-COR, USA). The daily plant weight (biomass) gain was determined based on the difference between the pot weights on the morning of two consecutive days, when the drainage from the pot (following an thorough-irrigation event) had finished. Agronomic WUE, defined as the ratio between the plant weight gain and the amount of water transpired. The WUE of each line was determined by fitting a linear curve for the cumulative plant weight gain during the well-irrigated stage vs. cumulative water transpiration (calculated daily based on the difference between pot weights before dawn and in the evening). Soil relative volumetric water content (SWC) was measured using the EC-5 soil moisture sensor combined with the 'ProCheck’ interface reader (Decagon Devices, Pullman, WA. USA). The SWC under well-irrigated and drought conditions were 80% and 30%, respectively. Under full field capacity, the SWC is equal to 80%, and hence 30% of SWC is equivalent to ~38% field capacity. The 30% SWC was reached within 10–15 days after the experiment was started and the flag leaves were fully expanded at this time. Leaf relative water content (RWC) was collected at 12:00 – 14:00 on days when the water content in the pots reached the specified soil water content levels. Measurement data were collected from a single leaf from each plant. RWC was evaluated using the following protocol: leaf fresh weight (FW) was immediately recorded, then leaves were soaked for 8 h in 5 mM CaCl solution at room temperature in the dark to record the turgid weight (TW). Total dry weight (DW) was recorded after drying these leaves at 70°C to a constant weight. Relative water content (RWC) was calculated as (FW - DW/TW - DW) × 100.

### Preparation of total RNA and cDNA for transcriptome sequencing

Total RNA was extracted with the TriZol RNA extraction kit from leaves of single B1K2 and B1K30 plants at the fifth day of water deficit period (Additional file 1: Figure S1). The total RNAs extracted from two stressed samples of both B1K2 and B1K30 ecotypes were quality checked and pooled together for ds cDNA synthesis and cDNA normalization. The quality test, ds cDNA synthesis and cDNA normalization processes were carried out by Evrogen (Evrogen Lab, http://www.evrogen.com) using the following steps. The ds cDNA synthesis of pooled samples was performed with the SMART method [57]. First strand cDNA synthesis was performed using 5 μl reaction mixture containing 0.3 μg of pooled total RNA, 10 pmol SMART Oligo II oligonucleotide (5′-AAGCAGTGGTATCAACGCAGAGTACGCrGrGrG-3′) and 10 pmol CDS-T22 primer (5′-AGCAGTGGTATCAACGCAGAGTTTTTGTTTTTTTCTTTTTTTTTTVN-3′). The ds cDNA synthesis was performed by long-distance PCR [58]. Finally, amplified cDNA PCR products were purified using QIAquick PCR Purification Kit (QIAGEN, CA) and concentrated by ethanol precipitation. The synthesized cDNA libraries were normalized using duplex-specific nuclease (DSN) normalization method [59] with the Evrogen Trimmer kit [60]. Finally, the normalized cDNA libraries were first diluted by adding 30 μl milliQ water and amplified in in 50 μl PCR reaction containing 1 μl diluted cDNA, 1 x Advantage 2 reaction buffer (Clontech), 200 μM dNTPs, 0.3 μM SMART PCR primer and 1 x Advantage 2 Polymerize mix (Clontech). A total of 18 PCR cycles each involving 95°C for 7 seconds, 65°C for 20 seconds; 72°C for 3 minutes was carried out.

### 454 transcriptome sequencing

The 454 GS FLX Titanium library construction, emPCR and sequencing were performed by Macrogen (Macrogen Inc., Seoul, Korea). After fragmentation of normalized cDNA libraries using nebulization, the quality of the libraries was checked with an Agilent 2100 Bioanalyzer. The libraries were sequenced on one full-plate (half-plate for each sample) using a 454 GS FLX Sequencer and Titanium Chemistry protocol.

### *De novo*assembly of transcriptome reads

A *de novo* assembly of 454 transcriptome sequence raw reads was carried out using *Newbler* v2.6 [61]. The overall summary of the workflow used for the *de novo* assembly of the transcriptome reads is indicated Additional file 1: Figure S1. As separate *de novo* assembly (of B1K2 and B1K30) was performed using the following parameters: -cdna (to assemble transcripts), -ml 90% (minimum overlap read length, default 40 bp), -urt (to extend contigs using the ends of single read) and -v (a fasta file database for trimming of the contaminants: adapters, primers and poly-A/T). The trimming database was developed based on contaminant information obtained from trial assemblies and contains a list of all used adapters and primers with their isoforms identified in the trial assemblies. To trim out poly-A/T, we also included a list of poly-As that contains >10 A in the trimming database. For the combined *de novo* assembly of B1K2 and B1K30 reads (B1K assembly), we used the same parameters as for the *de novo* assembly except for the following settings -ml 95%, -mi 95 (minimum overlap% identity for pairwise alignment, default 90) and -minlen 30 (minimum length of reads for assembly, default 20 bp). The assembled isotigs (which are equivalent to the transcripts) and trimmed singletons were further cleaned by SeqClean [62] using the following two databases: database of adapters and primers sequences used for cDNA library preparation, and database of vector sequences [63] to screen for contaminant vectors.

### Construction of a nonredundant barley full-length cDNA set

We downloaded 28,620 *Hordeum vulgare* full-length cDNAs (Hv. fl-cDNA) from the NCBI database: accession numbers AK248134 to AK253139 for 5,006 fl-cDNA [38] and AK353559 to AK377171 for 23,614 Hv. fl-cDNAs [37]. These 28,620 Hv. fl-cDNA sequences were clustered into 23,356 nonredundant (NR) sequences with a minimum of 100 bp length using CD-HIT-EST [64] with parameters: -c 0.98 and -n 9. The clustered NR Hv. fl-cDNAs were used as a reference for reference-based transcriptome mapping and for homologous gene search.

### Reference-based assembly of transcriptome reads

To identify SNPs within wild barley ecotypes and among wild and cultivated barley, high quality and clustered reads of B1K2 and B1K30 reads were mapped against NR Hv. fl-cDNAs, and B1K2 reads against PUTs of B1K30 sequences using Bowtie-2 v2-2.0.0-beta6 [65]. The overall summary of the workflow used for the reference-based mapping of the transcriptome reads is indicated in Additional file 1: Figure S1. B1K2 and B1K30 reads used for mapping were selected as follows. Initially, *de novo* assemblies were performed on B1K2 and B1K30 reads using Newbler v2.6 [66] (used parameters: -cdna, -urt, -mi 95, -ml 95% and -minlen 45). From the assembly output files, assembled (fully and partially) and singleton reads were identified and extracted using Roche 454’s *fnafile* utility. The extracted reads were further clustered into nonredundant reads using USEARCH/UCLUST program v5.1 [67]. Clustered reads were then cleaned using SeqClean program for contaminant vectors [63] and adapters and primers. Finally the cleaned and nonredundant reads were mapped to the indexed reference using Bowtie-2, using the '–local’ alignment mode. The same parameters were also used to map B1K2 high quality reads onto B1K30 sequences (NR isotigs and true singletons). In addition to Bowtie-2, we also used other two different assembly/SNP calling tools: BWA-SW implemented in BWA (Burrow-Wheeler Aligner) v0.5.9 [68] using –z 100 (Z-best heuristics, default =1) to increase the accuracy of the alignment, and GSMapper (Newbler v2.6) from Roche 454 [66]. The B1K reads were also mapped onto the filtered chromosomal barley reference genome, *Hordeum vulgare* 030312 v2.16 from ENSEMBL release 16 and barley HC genes CDS (version: MIPS 23 March 2012) [23] using Bowtie-2.

### SNP identification and annotation

To select the best SNP identification approach, we first compared the three SNP calling tools: Bowtie-2, BWA-SW and GSMapper using B1K2 reads mapped against Hv. fl-cDNA. After selecting Bowtie 2 for mapping, SNP identification was performed using SAMtools v0.1.18 [69] as follows. From sorted BAM files of B1K2 and B1K30 assemblies, raw SNPs were called by *mpileup* utility of SAMtools using the following parameters: -D (output per-sample read depth), -g (compute genotype likelihoods and output them in the binary call format [BCF]), -u (same as -g but uncompressed BCF), and –I (without INDEL calling). Called SNPs were further filtered by *varfilter* utility of *bcftools* using parameter -D (maximum read depth) of 1000 and a minimum depth of two reads to generate raw SNPs. From raw SNPs, high quality SNPs were further identified based on very stringent filtering criterion that a minimum of eight reads in which four reads each had to support both reference and variant nucleotides (alleles). This SNPs filtering approach was applied to discover high quality SNPs from mapping of B1K2 NR reads against B1K30 transcripts and the NR reads of both ecotypes against barley fl-cDNA and HC genes. For SNP annotation, we however used less stringent filtering criterion to reduce the loss of information, and hence SNPs with ≥8x coverage and a minimum of two reads supporting each allele (reference and variant) were used. Using this approach, SNPs discovered from mapping of B1K2, B1K30 and B1K reads against chromosomal barley reference genome were annotated using snpEff v3.1 [70].

### Estimation of the population recombination parameter, 4*N*_*e*_*r*

The population recombination parameter, 4*N*_*e*_*r* across barley chromosomes was estimated using a Bayesian reversible-jump MCMC scheme of the rhomap program included in the LDhat package v2.1 [71] using the following parameters: cross-over model, 1 million iterations, 1 million burn-ins and 100,000 samples. We used filtered SNP data (minimum depth =8x) from our transcriptome sequences (B1K2 and B1K30 SNPs) and wild barley (B1K4) genome sequence [23] to estimate the recombination rates.

### Identification of homologous genes

We compared each of our assemblies (B1K2, B1K30 and B1K) against NR Hv. fl-cDNA and 70,144 barley ESTs unigenes (HarvEST v1.83 assembly 36, http://harvest.ucr.edu/) using MegaBLAST with the following parameters: -p 95 -r 1 -q -2 -G 1 -E 2 and an E-value of <10^-10^. Blast-hits with an alignment length of ≥50 bp were filtered from the MegaBLAST tabular outputs and reciprocal blast-hits (RBHs) or were identified using PERL scripts [72]. We applied the same approaches to identify homology for our unique transcripts and to identify how many are annotated in the 26,159 HC genes CDS (version: MIPS_23Mar12) [21]. Homologous genes were identified in annotated proteins from other fully sequenced grass species: *Brachypodium distachyon* [73] (Brachypodium Genome v1.2, ftp://ftpmips.helmholtz-muenchen.de/plants/brachypodium/v1.2/), *Oryza sativa* [74] (MSU6, ftp://ftp.ensemblgenomes.org/pub/plants/release-11/fasta/oryza_sativa/pep/), *Sorghum bicolor* [75] (Sbi1_4, filtered model, http://genome.jgi-psf.org/Sorbi1/Sorbi1.download.ftp.html), *Zea mays* [76] (ZMB73_4a.53, filtered model, http://www.maizesequence.org/index.html) and *Arabidopsis thaliana* [77] (TAIR10, ftp://ftp.arabidopsis.org/home/tair/Proteins/). B1K2, B1K30 and B1K sequences were compared against all databases using BLASTX and outputs were filtered for hits with ≥75% similarity and an alignment length of ≥33 amino acids. RBH were identified from filtered hits using PERL scripts [72].

### Identification of stress- and other trait-related genes from 454 transcripts

Confirmed and putative stress related genes such as aquaporin, dehydrin, *ABC* (ATP-binding cassette subfamily G, ABCG or *Eibi*), *ERD* (Early Response to Dehydration stresses), *LEA* (Late Embryogenesis Abundance), *HSP* (Heat Shock Protein) in barley, wheat, sorghum, maize, rice and *Arabidopsis* were searched in the literature and downloaded from the Uniprot and NCBI databases. 481 protein sequences of these stress-related genes were compared with B1K2 and B1K30 sequences using BLASTX with an E-value of 10^-6^. Hits with similarity ≥75% and an alignment length of ≥33 amino acids were further filtered from the BLASTX tabular outputs, and RBH were identified from the filtered outputs using PERL scripts. Similarly, to identify wild barley transcripts that were homologous to already identified rice trait genes, we downloaded the list of all genes from Oryzabase [78] from http://www.shigen.nig.ac.jp/rice/oryzabase/. Rice trait genes homologous to our 454 sequences were then identified from RBH (similarity ≥75% and an alignment length of ≥33 amino acids) results to rice genome by their IDs.

### Transcription factors identification from 454 transcripts

Protein sequences of barley, *Brachypodium distachyon*, rice (*O. sativa* Japonica), sorghum, maize, wheat (*Triticum aestivum*) and *Arabidopsis thaliana* transcription factors were downloaded from Peking University Plant Transcription factors Database [79] at http://planttfdb.cbi.pku.edu.cn/. Sequences were separated into two groups (barley and the rest) and the RBH approach was used for the identification of putatively orthologous wild barley transcription factors using BLASTX and E-value of 1e-20 and further filtering for hits with similarity ≥85% and an alignment length of ≥33 amino acids.

### Functional annotation of putative unique transcripts

The functional annotation and classification of clustered wild barley was carried out with Blast2GO v2.5 [80]. The unique transcripts were compared against the NCBI non-redundant (NR) and Swiss-Prot protein databases using BLASTX with an E-value of 1e-06, and GO terms were mapped to the obtained hits. The mapped GO terms were initially annotated with the default annotation threshold (55) and un-annotated sequences were further annotated at a threshold of 45. GO annotations were further enriched and refined using the two methods implemented in Blast2GO, Augment Annotation by Annex (ANNEX) [81] and GO-Slims [82] methods. After the GO annotations were refined by GO-slim for plant, GO terms were functionally categorized into biological process, molecular functions and cellular components at level 2. The InterPro scan annotation was also performed using Blast2GO program to identify protein family and domain associated with wild barley transcripts.

### Gene prediction

We estimated the number of barley HC genes captured by our assembled transcripts using three different homology-based search approaches: one-to-many, RBH and BLASTX. For BLASTX hit search, we used BLASTX parser pipeline (http://www.atgc.org/SeqsExtractor/) to extract Hv. HC CDS homologous CDS from our transcripts. Furthermore, OrfPredictor [83] was used to predict the CDS from the *de novo* assembled unique transcripts.

### Availability of supporting data

Raw 454 sequence data were submitted to the NCBI short sequence read archive (Accession number: SRX319401 and SRX319413). Putative unique transcripts, SNP calls and annotation are available from LabArchives (DOI:10.6070/H4R20ZB1).

## Electronic supplementary material

Additional file 1: Figure S1: Summary of the workflow used for 454 transcriptome sequence analysis. Two mapping approaches were used: (i) *de novo* mapping of 454 reads (left side) for the assembly of PUTs, and (ii) reference-based mapping of all duplicate (right with broken line boxes) and non-duplicate (right with dot line boxes) 454 reads for SNP discovery. SNPs called from chromosomal barley reference genome (WGS – whole genome sequence) were annotated. **Figure S2.** Effect of 454 transcript length on homology identification in cultivated barley sequences. Homology search based on one-way (one-to-many) and RBH approaches. **Figure S3.** Annotation of wild barley 454 transcripts. (A) Top-hit species distribution based on BLASTX hit against NCBI’s *nr* database. The order is based on top-hit of B1K and only species with over 1% are shown while the rest is included in the 'others’. (B) Effect of query sequence (PUT) length on the annotations of *de novo* assembled B1K2 and B1K30 PUTs based on annotation against NCBI’s *nr* (solid lines, left) and Swiss-Prot (dash lines, right) databases. **Figure S4.** Functional annotation of wild barley 454 transcripts and barley genes based on Gene Ontology (GO) assignment and classification. GO terms assignment to wild barley 454 sequences and classification into three categories (biological process, molecular function and cellular components) are based on BLASTX search against Swiss-Prot database. 'Barley Affy’ GO terms assigned to cultivated barley sequences from Affymatrix Barley Genome Array. GO slim analysis at level 2 applied to both sequences. **Figure S5.** Protein sequence alignments of selected transcripts longer than their orthologous barley genes. Four longer PUTs and their orthologous barley genes and genes with full length from UniProt are aligned to show how the transcripts can be used to improve barley genome annotation: (A) B1K2_isotig00096; (B) B1K2_contig00374; (C) B1K_contig00468; and (D) B1K_isotig01338. **Figure S6.** Genome-wide distribution of SNPs identified among wild and cultivated barley. The Circos histogram shows the frequency of all identified SNPs per 10 kb. For the display, the maximum SNPs frequencies are set to 50. **Figure S7.** Distribution of SNPs identified within wild barley and among wild and cultivated barley. (A) Frequency distribution of SNPs identified within wild barley PUTs and among PUTs and Hv. fl-cDNA. (B) Frequency distribution of SNPs identified among wild barley PUTs and Hv. HC genes. **Figure S8.** Distribution of sSNP and nsSNPs in selected barley genes. Proportions and numbers of sSNPs and nsSNPs in B1K2 and B1K30 are given for genes with a minimum of five total SNPs and at least one nsSNP in the B1K2 ecotype. **Figure S9.** Correlation between SNP density and population recombination parameter. There is no significant correlation (Pearson’s r = -0.03, p =0.12) between SNP density per kb and average recombination rate per kb. (PDF 8 MB)

Additional file 2: Table S1: Comparison of wild barley 454 transcripts and Hv fl-cDNA sequences against different plant genomes. **Table S4.** Summary of stress-related candidate genes identified from functional annotation of B1K2 and B1K30 transcriptome sequences. **Table S5.** CDS predicted from assembled unique transcripts based on comparison against Hv. HC CDS and using OrfPredictor. **Table S6.** Summary of SNPs shared among three wild barley ecotypes. **Table S7.** List of top 30 barley genes with high number of SNPs with moderate and high effects (nsSNP+). Top 30 genes were selected based on nsSNP + in B1K2. (DOCX 41 KB)

Additional file 3: Table S2: Summary of homologous searches. Homology searches of B1K2, B1K30 and B1K PUTs against each other, different barley and selected cereals sequences and different protein databases. '0’: PUTs without hit and '1’: PUTs with hit. **Table S3.** Summary of homologous searches against stress-related genes and plant transcription factors. RBH-based homology searches of B1K2 and B1K30 unique transcripts against selected stress-related genes and transcription factors from barley, selected grasses and *Arabidopsis thaliana*. (XLSX 84 KB)

## Abbreviations

B1K2 (B1K-2-8): Desert wild barley ecotype
B1K30 (B1K-30-9): Mediterranean/costal wild barley ecotype
B1K4 (B1K-04-12): Wild barley ecotype from Ein Prat, Israel
B1K: *de novo* assembly from B1K2 and B1K30 reads
Hv. Fl-cDNA: Barley full length cDNA
Hv. HC: Barley high-confidence
SNP: Single nucleotide polymorphism
WGS: Whole genome sequence
RBH: Reciprocal (bi-directional) blast hit
SD: Standard deviation.
