# Supplementary figures and images for: Transcriptome sequencing of two wild barley (Hordeum spontaneum L.) ecotypes differentially adapted to drought stress reveals ecotype-specific transcripts
Source: BMC Genomics. 2014 Nov 19;15(1):995. doi: 10.1186/1471-2164-15-995 (PMC4251939; doi:10.1186/1471-2164-15-995)

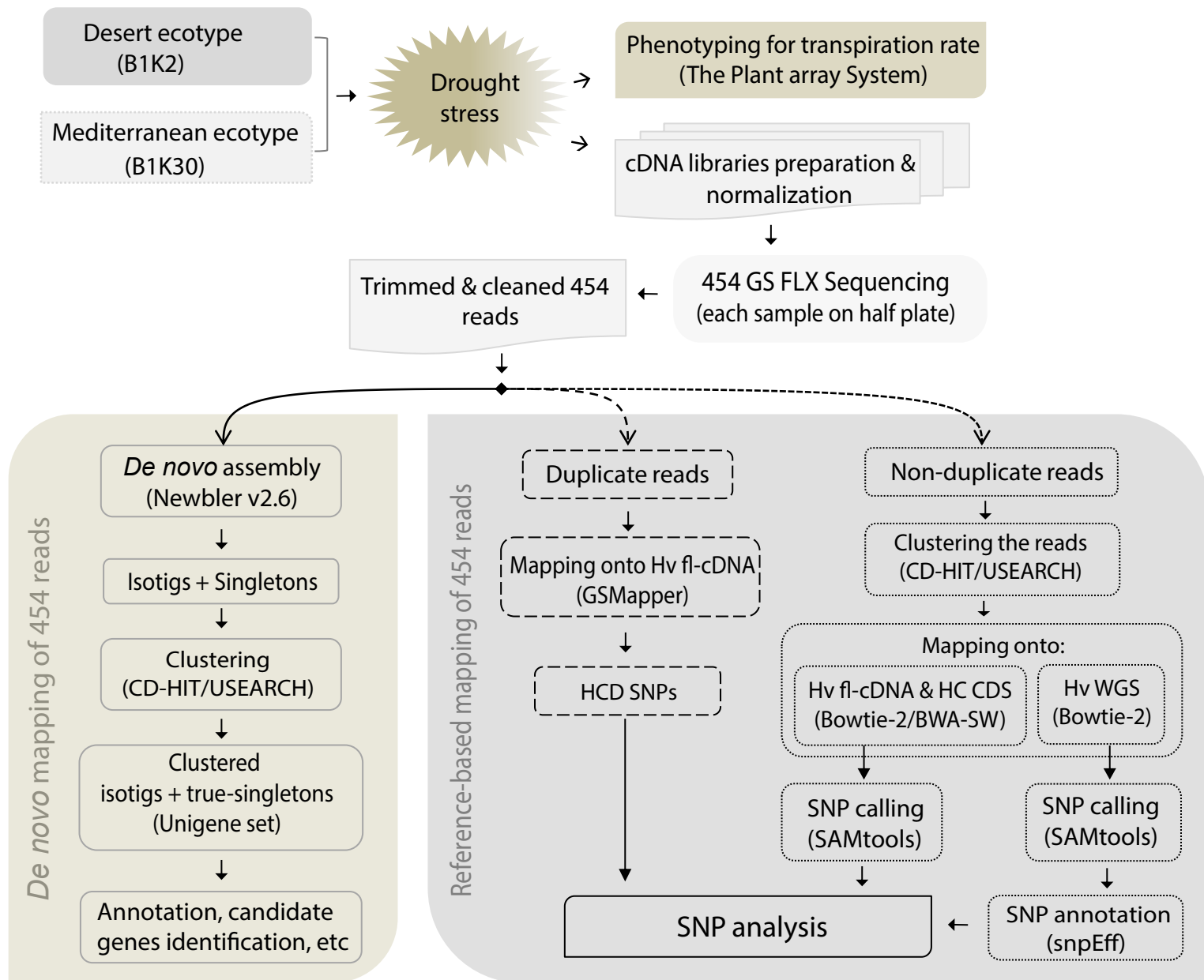

Figure S1

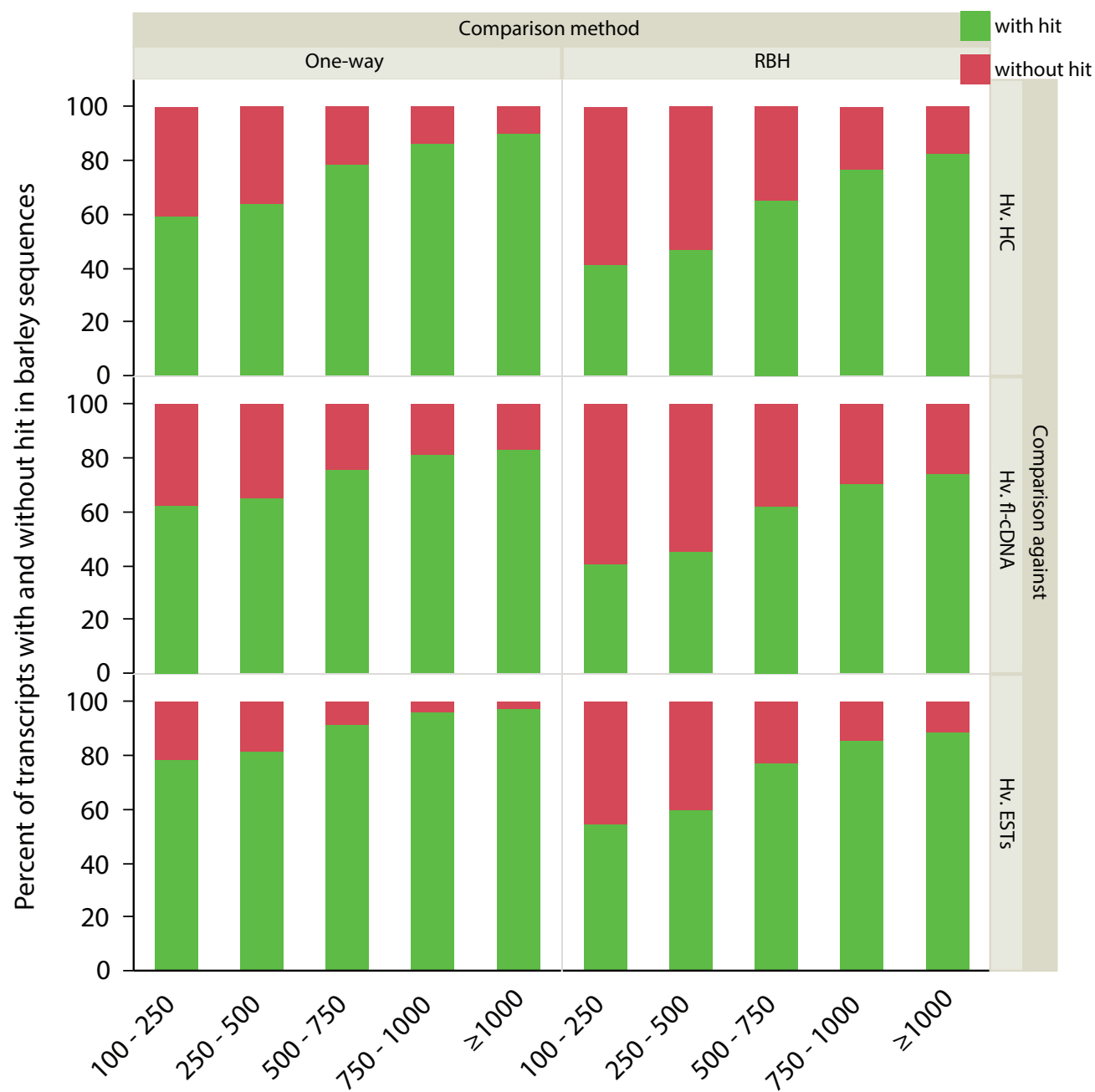

Figure S2

(A)

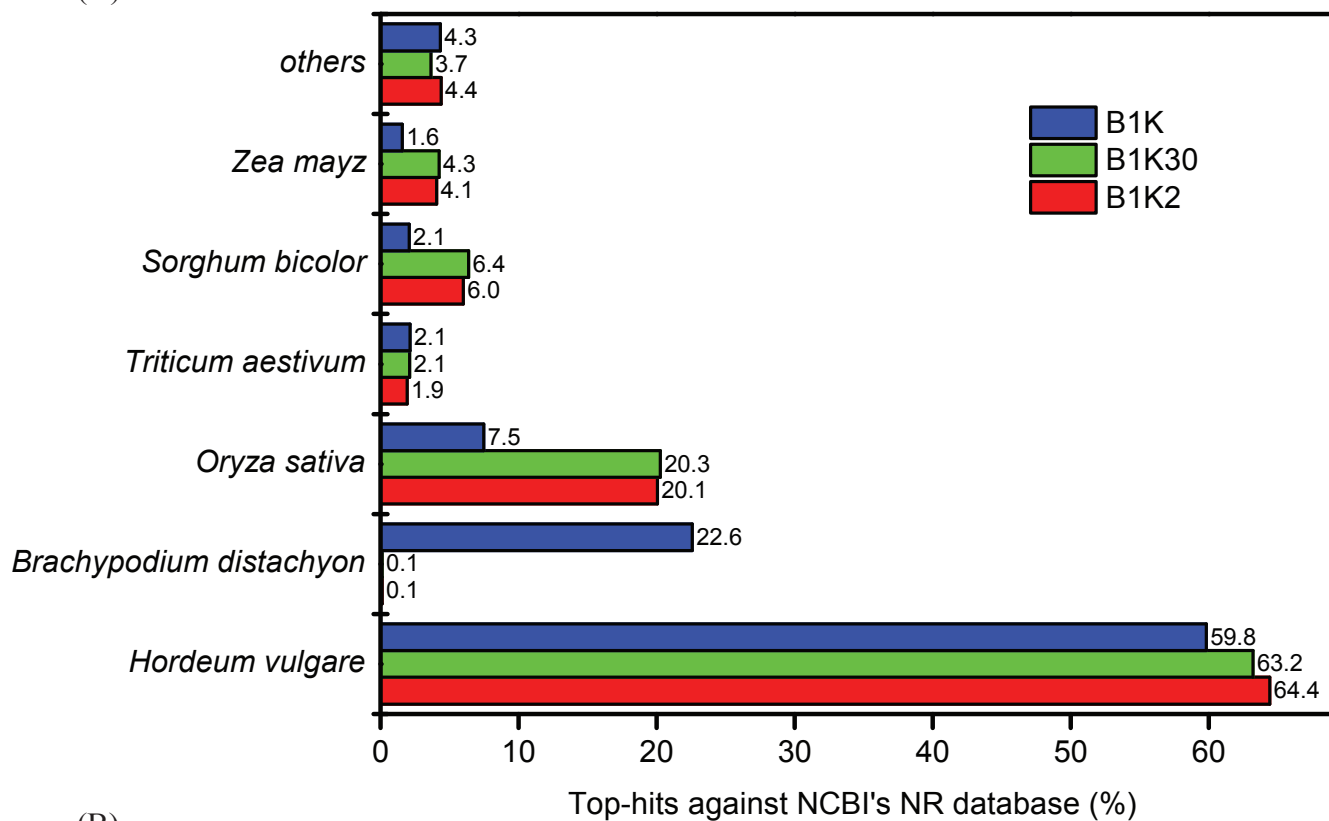

(B)

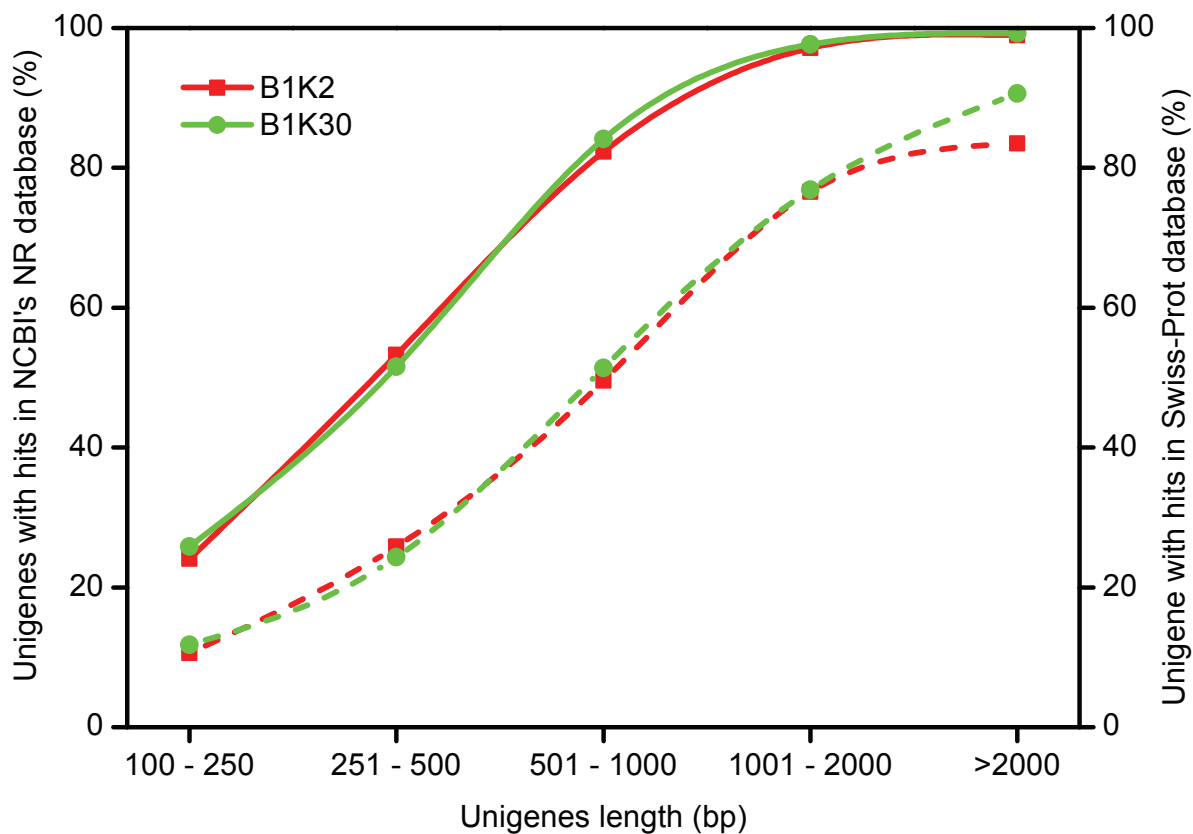

Figure S3

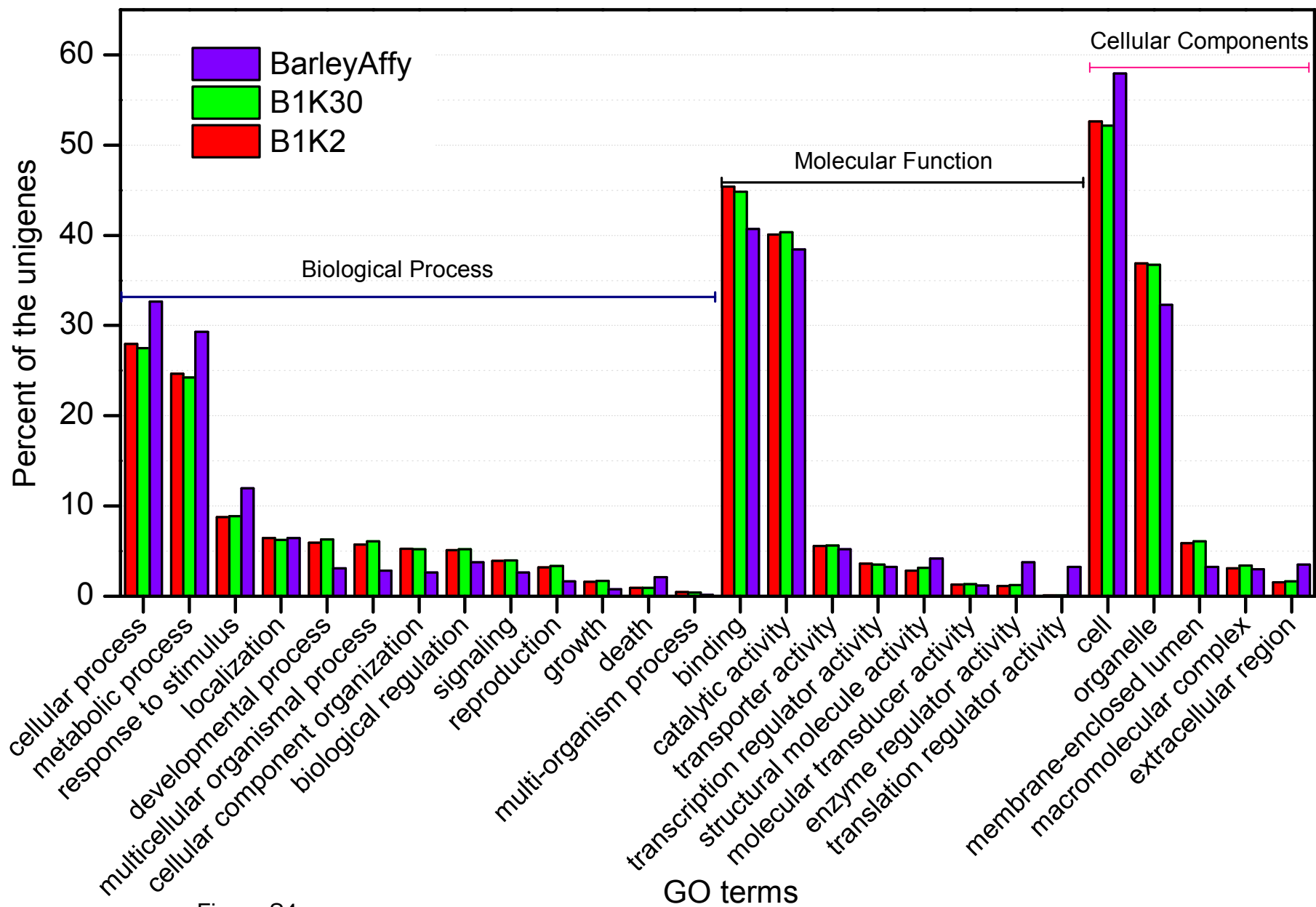

Figure S4

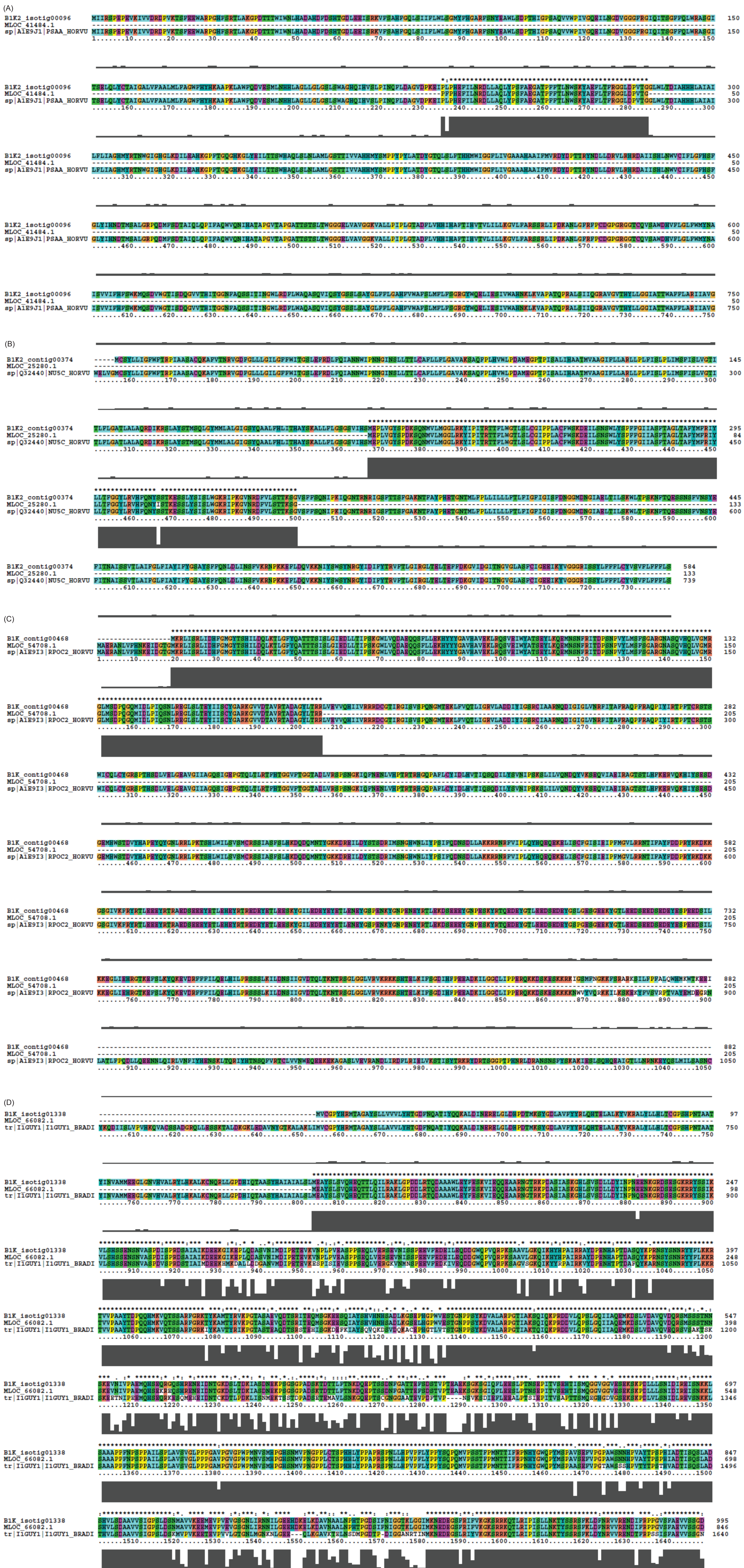

Figure S5

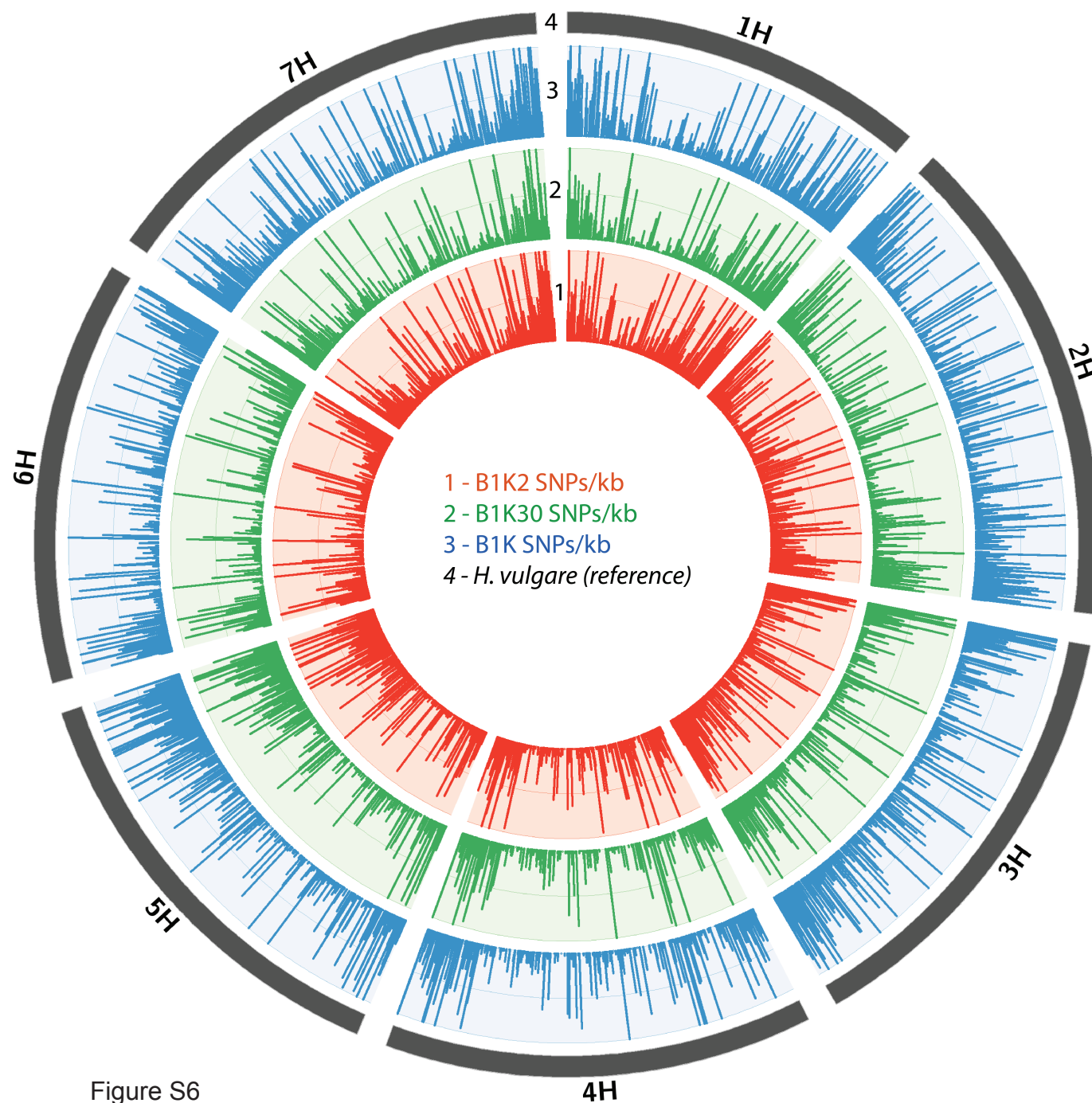

Figure S6

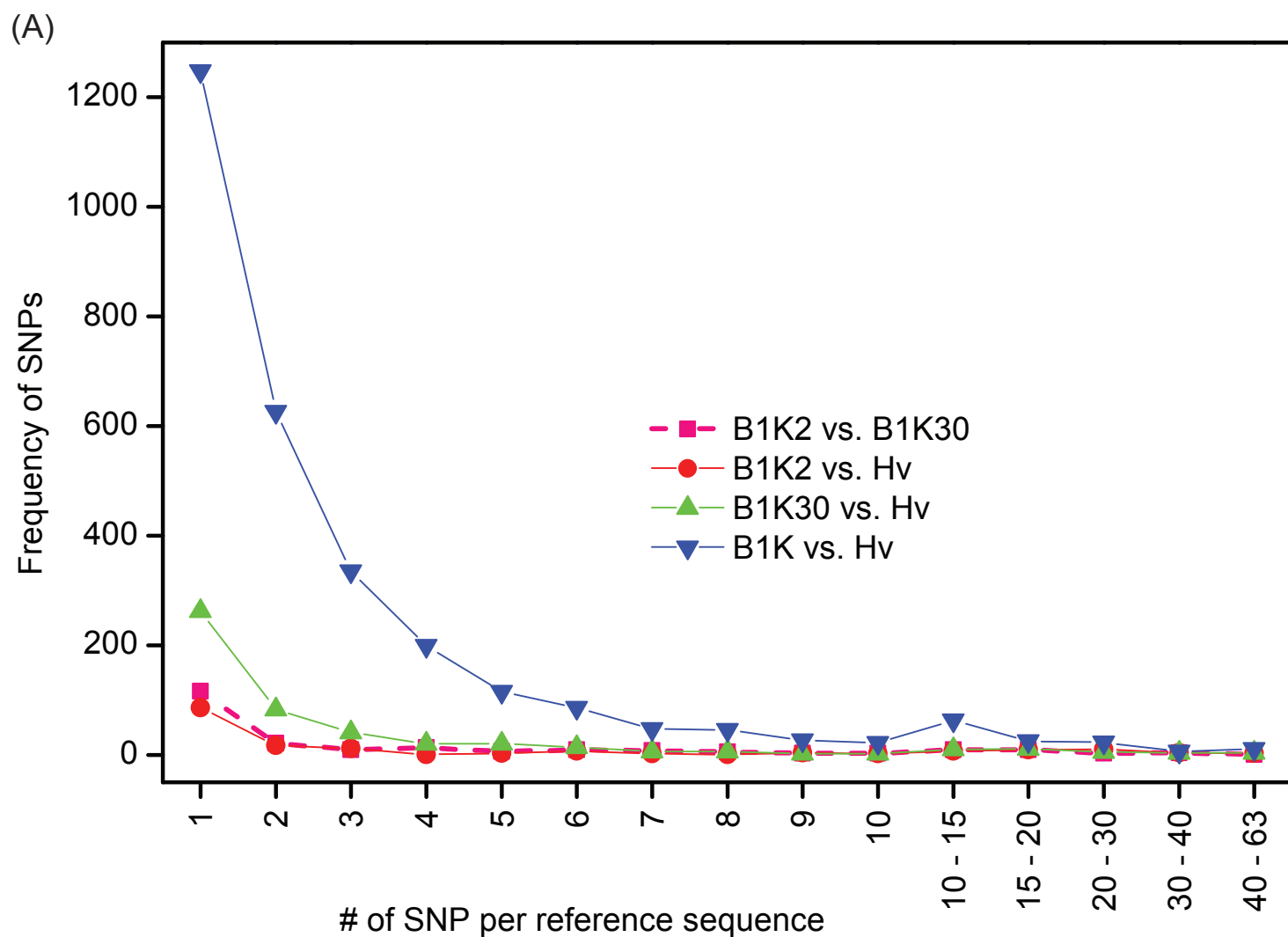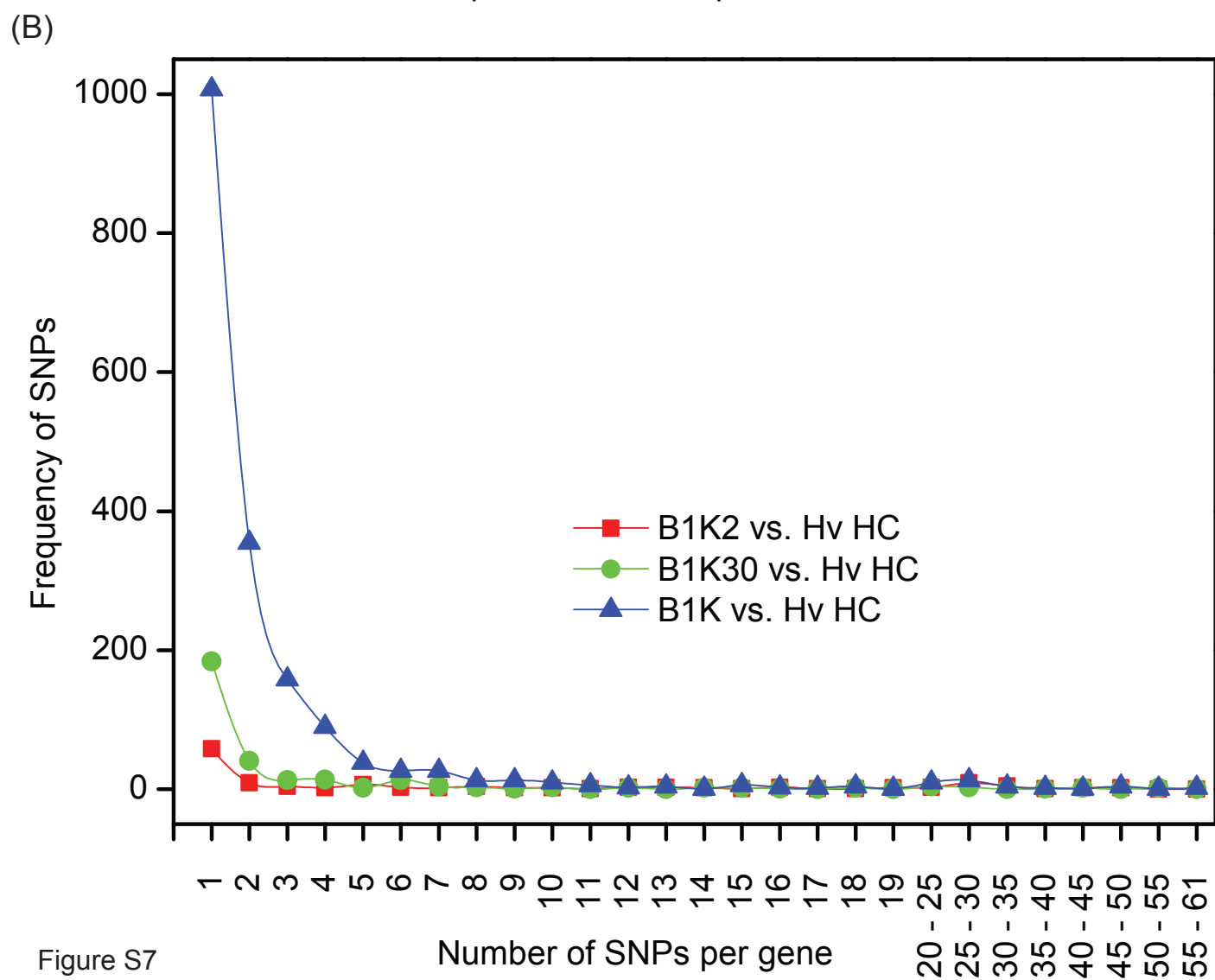

Figure S7

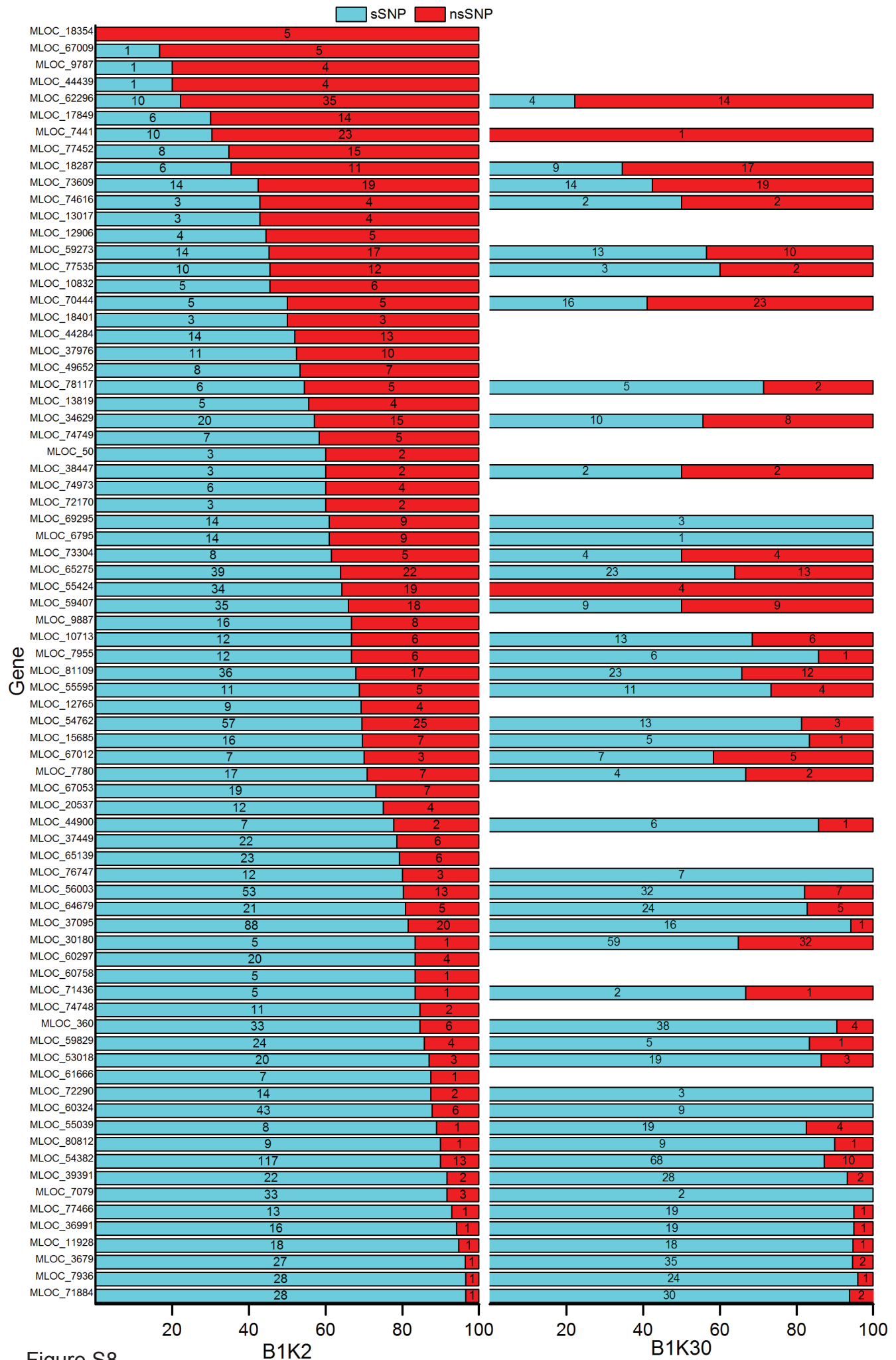

Figure S8

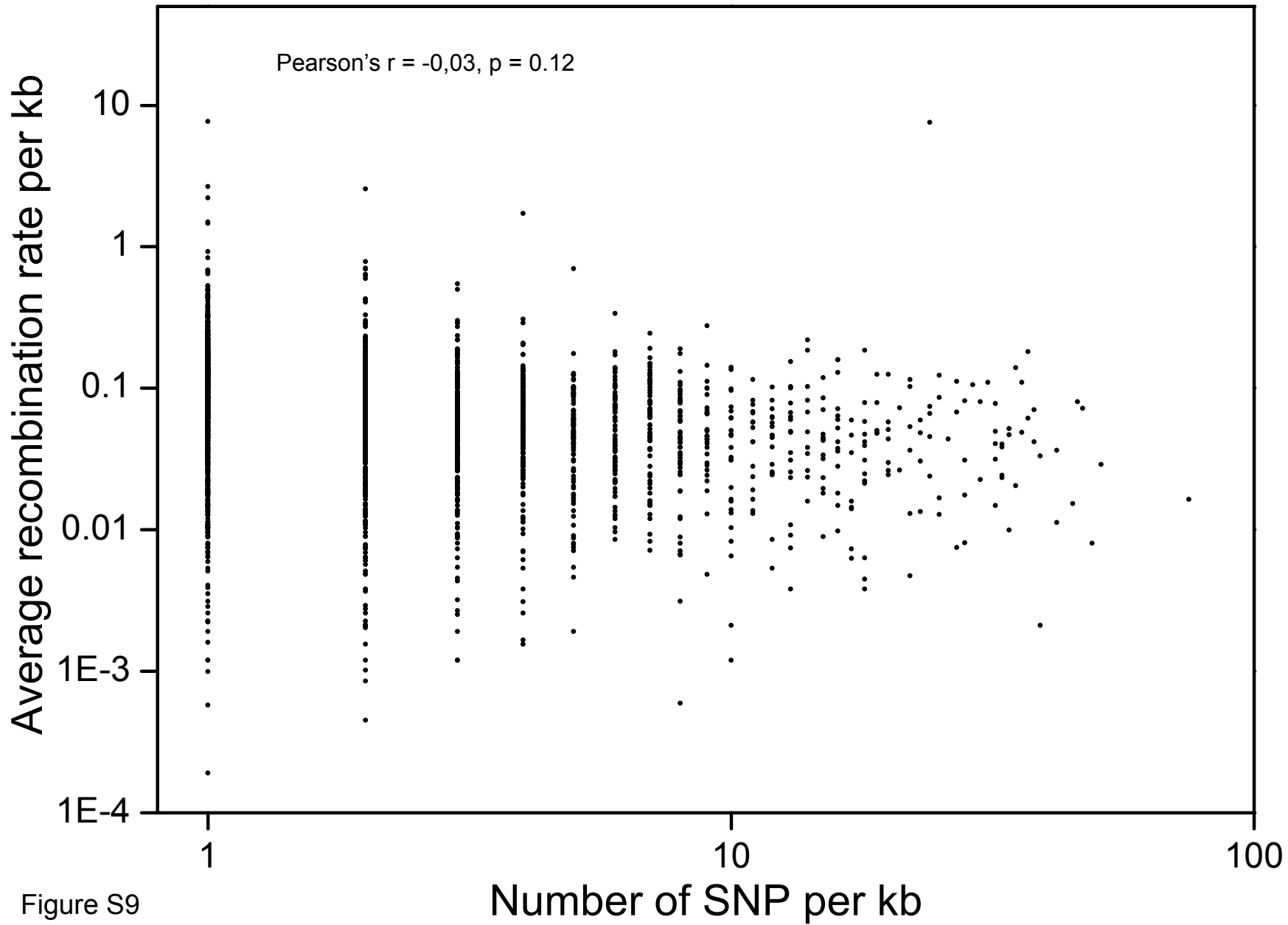

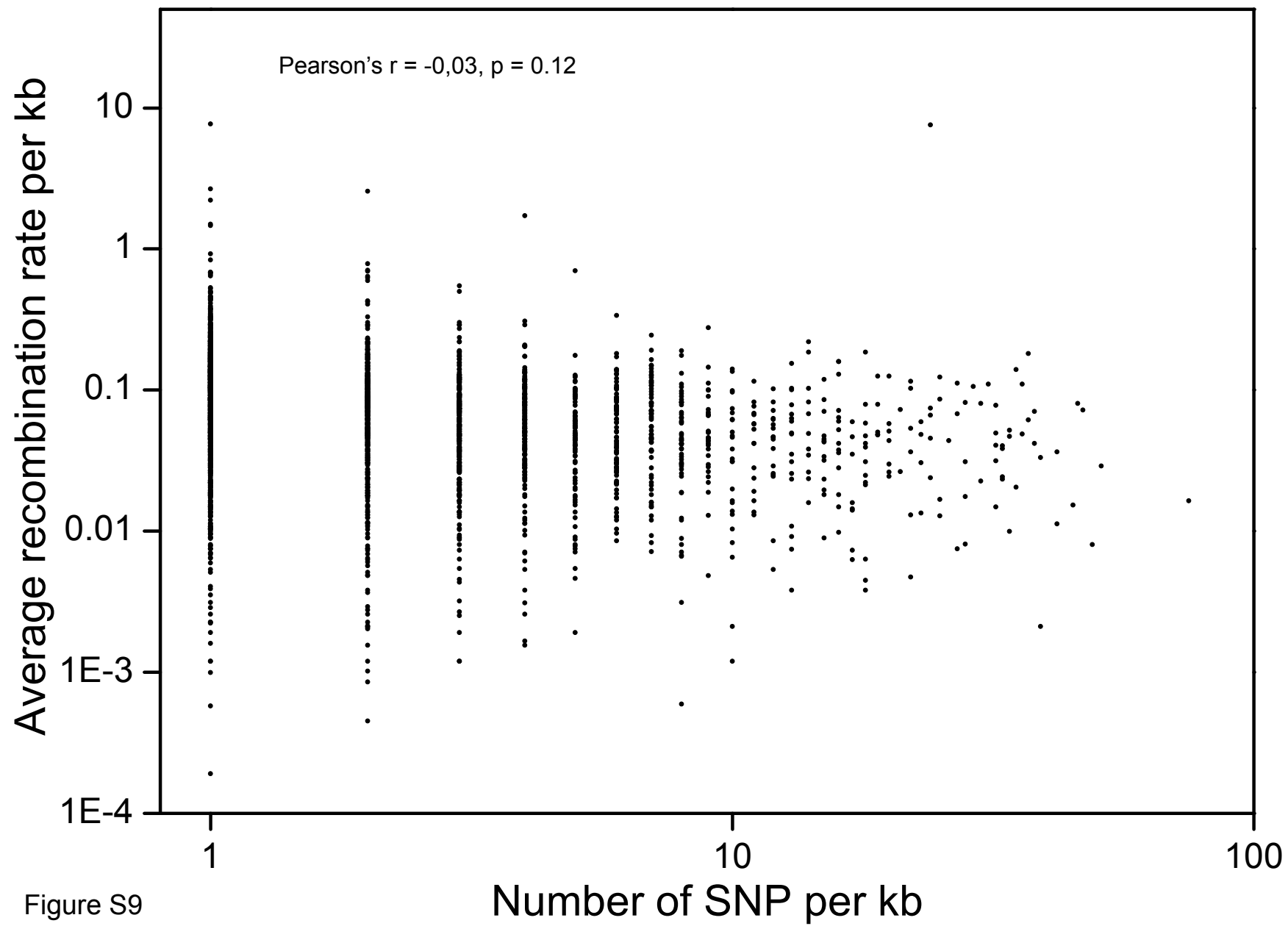

Supplement: Supplementary file 1 — Additional file 1: Figure S1: Summary of the workflow used for 454 transcriptome sequence analysis. Two mapping approaches were used: (i) de novo mapping of 454 reads (left side) for the assembly of PUTs, and (ii) reference-based mapping of all duplicate (right with broken line boxes) and non-duplicate (right with dot line boxes) 454 reads for SNP discovery. SNPs called from chromosomal barley reference genome (WGS – whole genome sequence) were annotated. Figure S2. Effect of 454 transcript length on homology identification in cultivated barley sequences. Homology search based on one-way (one-to-many) and RBH approaches. Figure S3. Annotation of wild barley 454 transcripts. (A) Top-hit species distribution based on BLASTX hit against NCBI’s nr database. The order is based on top-hit of B1K and only species with over 1% are shown while the rest is included in the 'others’. (B) Effect of query sequence (PUT) length on the annotations of de novo assembled B1K2 and B1K30 PUTs based on annotation against NCBI’s nr (solid lines, left) and Swiss-Prot (dash lines, right) databases. Figure S4. Functional annotation of wild barley 454 transcripts and barley genes based on Gene Ontology (GO) assignment and classification. GO terms assignment to wild barley 454 sequences and classification into three categories (biological process, molecular function and cellular components) are based on BLASTX search against Swiss-Prot database. 'Barley Affy’ GO terms assigned to cultivated barley sequences from Affymatrix Barley Genome Array. GO slim analysis at level 2 applied to both sequences. Figure S5. Protein sequence alignments of selected transcripts longer than their orthologous barley genes. Four longer PUTs and their orthologous barley genes and genes with full length from UniProt are aligned to show how the transcripts can be used to improve barley genome annotation: (A) B1K2_isotig00096; (B) B1K2_contig00374; (C) B1K_contig00468; and (D) B1K_isotig01338. Figure S6. Genome-wide di [file 12864_2014_6701_MOESM1_ESM.pdf]
