# Supplementary material for: Transcriptome sequencing of two wild barley (Hordeum spontaneum L.) ecotypes differentially adapted to drought stress reveals ecotype-specific transcripts
Source: BMC Genomics. 2014 Nov 19;15(1):995. doi: 10.1186/1471-2164-15-995 (PMC4251939; doi:10.1186/1471-2164-15-995)
Supplement: Supplementary file 2 — Additional file 2: Table S1: Comparison of wild barley 454 transcripts and Hv fl-cDNA sequences against different plant genomes. Table S4. Summary of stress-related candidate genes identified from functional annotation of B1K2 and B1K30 transcriptome sequences. Table S5. CDS predicted from assembled unique transcripts based on comparison against Hv. HC CDS and using OrfPredictor. Table S6. Summary of SNPs shared among three wild barley ecotypes. Table S7. List of top 30 barley genes with high number of SNPs with moderate and high effects (nsSNP+). Top 30 genes were selected based on nsSNP + in B1K2. (DOCX 41 KB) [file 12864_2014_6701_MOESM2_ESM.docx]

**Additional file 1:**

Format: PDF Size 137KB

**Table S1 Comparison of wild barley 454 transcripts and Hv fl-cDNA sequences against different plant genomes.**

| Barley data  (No. of sequences) | ^1^Genome Annotation | One-to-many | | RBH | |
| --- | --- | --- | --- | --- | --- |
|  |  | No. of PUTs | % of PUTs | No. of PUTs | %of PUTs |
| B1K2  (20,439) | Barley | 13402 | 65.6 | 10151 | 49.7 |
|  | Brachypodium | 10788 | 52.8 | 7203 | 35.2 |
|  | Rice | 9423 | 46.1 | 6217 | 30.4 |
|  | Sorghum | 8799 | 43.1 | 5768 | 28.2 |
|  | Maize | 8297 | 40.6 | 4953 | 24.2 |
|  | Arabidopsis | 3386 | 16.6 | 1703 | 8.3 |
| B1K30  (21,494) | Barley | 13632 | 63.4 | 9996 | 46.5 |
|  | Brachypodium | 10545 | 49.1 | 6976 | 32.5 |
|  | Rice | 9181 | 42.7 | 6048 | 28.1 |
|  | Sorghum | 8584 | 39.9 | 5613 | 26.1 |
|  | Maize | 8108 | 37.7 | 4765 | 22.2 |
|  | Arabidopsis | 3323 | 15.5 | 1646 | 7.7 |
| B1K  (28,719) | Barley | 17033 | 59.3 | 11859 | 41.3 |
|  | Brachypodium | 13404 | 46.7 | 8246 | 28.7 |
|  | rice | 11649 | 40.6 | 7158 | 24.9 |
|  | sorghum | 10835 | 37.7 | 6613 | 23.0 |
| Hv fl-cDNA (23,356) | Brachypodium | 15237 | 65.2 | 9578 | 41.0 |
|  | rice | 13073 | 56.0 | 3770 | 16.1 |
|  | sorghum | 12125 | 51.9 | 7013 | 30.0 |
|  | maize | 10193 | 43.6 | 7438 | 31.8 |
|  | Arabidopsis | 2907 | 12.4 | 1659 | 7.1 |
| ^1^Genome Annotation and database version (# of protein sequences): Barley HighConf genes MIPS 23Mar12 CDS (26,159), *Brachypodium distachyon* v1.2 (31,029), Rice MUS6 (68,619), Sorghum-filtered v1.4 (35,899), Maize-filtered ZmB73-4a.53 (53,764), and *Arabidopsis thaliana* TAIR10.3 (35,386). MegaBLAST cutoff: ≤1e-10, ≥ 75 %ID & ≥ 50 nt, and BLASTX cutoff: ≤1e-6 ≥ 75 %ID & ≥ 33 aa | | | | | |

**Table S4 Summary of stress-related candidate genes identified from functional annotation of B1K2 and B1K30 transcriptome sequences.**

| Candidate genes | B1K2 PUTs | B1K30 PUts |
| --- | --- | --- |
| ABC transporter | 104 | 93 |
| ABRE | 4 | 4 |
| Aldehyde dehydrogenase | 22 | 20 |
| Amino acid transport protein (AAP2) | 4 | - |
| AP2 | 10 | 9 |
| Aquaporin | 16 | 10 |
| Betaine aldehyde dehydrogenase | 10 | 4 |
| bHLH | 19 | 21 |
| bZIP | 16 | 14 |
| Calmodulin | 39 | 40 |
| CBF | 3 | 4 |
| CDPK | 17 | 17 |
| Dehydrin | - | 1 |
| DREB | 1 | 2 |
| ERD | 1 | 1 |
| ERF | 5 | 2 |
| Histone deacetylase | 17 | 17 |
| Homeodomain | 10 | 11 |
| HSF | 11 | 12 |
| HSP | 61 | 63 |
| MAPK | 28 | 19 |
| MYB | 46 | 42 |
| MYC | 1 | 3 |
| NAC | 9 | 11 |
| NADP | 46 | 45 |
| PEPC | 20 | 14 |
| Phospholipase | 23 | 38 |
| Polyamines | 20 | 22 |
| Proline | 25 | 32 |
| Pyruvate dehydrogenase | 21 | 19 |
| Stress responsive | 7 | 8 |
| SUMO | 10 | 12 |
| Superoxide dismutase | 7 | 8 |
| Trehalose | 10 | 10 |
| WRKY | 11 | 12 |
| Zinc finger | 185 | 241 |
| Total abiotic stress-related 454 transcripts | 839 | 881 |
| ABRE, ABA-responsive element; AP2, activator protein-2 ; bHLH, basic helix-loop-helix; bZIP, basic leucine zipper domain; CBF, C-repeat biding factor; CDPK, calcium-dependent protein kinase; DRE, dehydration-responsive element; HSF, heat shock factor; HSP, heat shock protein; MAPK, mitogen activated protein kinase; NAC, nascent polypeptide-associated complex; PEPC, phosphoenolpyruvate carboxylase; Polyamine (polyamine oxidase, S-adenosylmethionine, S-adenosylmethionine decarboxylase, S-adenosylmethionine synthase, spermidine, spermidine synthase, spermine and spermine synthase); SUMO, small ubiquitin-related modifier; Trehalase (trehalase, trehalose-phosphatase and trehalase-phosphate synthase). | | |

**Table S5 CDS predicted from assembled unique transcripts based on comparison against Hv. HC CDS and usinf OrfPredictor.**

|  | B1K2 (%) | B1K30 (%) | B1K (%) |
| --- | --- | --- | --- |
| Total clustered unique transcripts | 20,439 | 21,492 | 28,720 |
| CDS predicted based on one-to-many hits (≥ 50 bp): | | | |
| - Unique transcripts homologous to Hv. HC CDS | 13,402 (65.6) | 13,632 (63.4) | 17,033 (59.3) |
| - Hv. HC CDS homologous to unique transcripts | 11,497 (44.0) | 11,266 (43.1) | 13,211 (50.5) |
| CDS predicted based on RBH (≥ 50 bp): | | | |
| - Unique transcripts homologous to Hv. HC CDS | 10,151 (49.7) | 9,996 (46.5) | 11,859 (41.3) |
| - Hv. HC CDS homologous to unique transcripts | 10,660 (40.8) | 10,497 (40.1) | 12,226 (46.7) |
| CDS predicted based on BLASTX hit (≥ 70 bp): | | | |
| - Unique transcripts homologous to Hv. HC CDS | 13,722 (67.1) | 13,688 (63.7) | 17,675 (61.5) |
| - Hv. HC CDS homologous to unique transcripts | 10,129 (38.7) | 9,871 (37.8) | 11,735 (44.9) |
| - CDS fully covered (100% of Hv. HC CDS) | 597 (2.9) | 488 (2.3) | 796 (2.8) |
| - CDS ≥ 90% of Hv. HC CDS | 984 (4.8) | 754 (3.5) | 1,328 (4.6) |
| - CDS ≥ 85% of Hv. HC CDS | 1,188 (5.8) | 926 (4.3) | 1,630 (5.7) |
| - Unique transcripts without hit in Hv. HC genes | 6,717 (32.9) | 7,804 (36.3) | 11,045 (38.5) |
| CDS predicted by OrfPredictor | | | |
| - Unique transcripts with CDS | 20,348 (99.6) | 21,397 (99.6) | 28,549 (99.4) |
| - With CDS & homolog to Hv. HC genes | 13,722 | 13,684 | 17,604 |
| - With CDS but not homolog to Hv. HC genes | 6,626 | 7,713 | 10,945 |
| - Without CDS but homolog to Hv. HC genes | 0 | 2 | 71 |
| - Mean CDS length (bp) | 357 | 321 | 350 |
| - CDS ≥ 100 bp | 19,700 (96.8) | 20,597 (96.3) | 27,324 (95.7) |
| - CDS ≥ 500 bp | 3,895 (19.1) | 3,319 (15.6) | 5,530 (19.4) |
| - CDS ≥ 1 kb | 721 (3.5) | 469 (2.1) | 1,146 (4.0) |
| - CDS ≥ 85% of Hv. HC CDS | 1,112 (8.1) | 897 (6.6) | 1,512 (8.6) |
| - CDS equal with Hv. HC CDS | 449 (3.3) | 344 (2.5) | 584 (3.3) |
| - CDS longer than Hv. HC CDS | 320 (2.3) | 286 (2.1) | 456 (2.6) |
| CDS predicted from novel transcripts | **3,245** | **3,674** | **7,102** |
| - Novel transcripts with predicted CDS | 3,174 (98.7) | 3,600 (98.0) | 6,953 (97.9) |
| - CDS ≥ 100 bp | 2,774 (85.5) | 3,128 (85.1) | 6,045 (85.1) |
| - CDS ≥ 200 bp | 1,031 (31.8) | 998 (27.2) | 2,099 (29.6) |
| - CDS ≥ 500 bp | 19 (0.6) | 15 (0.4) | 38 (0.5) |

**Table S6 Summary of SNPs shared among three wild barley ecotypes.**

| **Reference (Morex) vs.** | **Private and shared SNPs:** | | **Remark** |
| --- | --- | --- | --- |
|  | **in number** | **in %** |  |
| B1K2 | 20741 | 52.45 | B1K2-specific SNPs |
| B1K2 vs. B1K30 | 9029 | 22.83 | SNPs found in both B1K2 & B1k30 |
| B1K2 vs. B1K4* | 5444 | 13.77 | SNPs found in both B1K2 & B1K4 |
| B1K2 vs. B1K30 vs. B1K4 | 4331 | 10.95 | SNPs found in all three ecotypes |
| ***B1K2 total SNPs*** | **39545** |  |  |
| B1K30 | 18311 | 50.83 | B1K30-specific SNPs |
| B1K30 vs. B1K2 | 9029 | 25.07 | SNPs found in both B1K30 & B1K2 |
| B1K30 vs. B1K4 | 4351 | 12.08 | SNPs found in both B1K30 & B1K2 |
| B1K30 vs. B1K2 vs. B1K4 | 4331 | 12.02 | SNPs found in all three ecotypes |
| ***B1K30 total SNPs*** | **36022** |  |  |
| *B1K4: SNPs data from wild barley ecotype used for barley genome sequencing project. | | | |

**Table S7 List of top 30 barley genes with high number of SNP with moderate and high effects (nsSNP+).** Top 30 genes were selected based on nsSNP+ in B1K2.

| Gene | Chr. | B1K2 | | B1K30 | | nsSNP+:sSNP | | Description |
| --- | --- | --- | --- | --- | --- | --- | --- | --- |
|  |  | sSNP | nsSNP+ | sSNP | nsSNP+ | B1K2 | B1K30 |  |
| MLOC_62296 | 5 | 10 | 35 | 4 | 14 | 3.50 | 3.50 | Unknown protein |
| MLOC_54762 | 2 | 57 | 25 | 13 | 3 | 0.44 | 0.23 | Chloride channel 1 |
| MLOC_7441 | 3 | 10 | 23 | 0 | 1 | 2.30 |  | Trimeric LpxA-like |
| MLOC_65275 | 3 | 39 | 22 | 23 | 13 | 0.56 | 0.57 | Nup98 protein/Peptidase S59, nucleoporin |
| MLOC_37095 | 5 | 88 | 20 | 16 | 1 | 0.23 | 0.06 | Ubiquitin-activating enzyme/NAD(P)-binding domain |
| MLOC_55424 | 4 | 34 | 19 | 0 | 4 | 0.56 |  | 60S acidic ribosomal protein P0, putative, expressed |
| MLOC_73609 | 5 | 14 | 19 | 14 | 19 | 1.36 | 1.36 | Saposin B domain-containing protein |
| MLOC_59407 | 3 | 35 | 18 | 9 | 9 | 0.51 | 1.00 | Vacuolar processing enzyme 3 |
| MLOC_59273 | 1 | 14 | 17 | 13 | 10 | 1.21 | 0.77 | Ribosomal protein L28e |
| MLOC_81109 | 1 | 36 | 17 | 23 | 12 | 0.47 | 0.52 | Chlorophyll a-b binding protein 3C-like |
| MLOC_34629 | 4 | 20 | 15 | 10 | 8 | 0.75 | 0.80 | O-methyltransferase |
| MLOC_77452 | 3 | 8 | 15 |  |  | 1.88 |  | Unknown protein |
| MLOC_17849 | 4 | 6 | 14 |  |  | 2.33 |  | Peptidyl-prolyl cis-trans isomerase |
| MLOC_44284 | 4 | 14 | 13 |  |  | 0.93 |  | Kinase interacting (KIP1-like) protein |
| MLOC_54382 | 1 | 117 | 13 | 68 | 10 | 0.11 | 0.15 | Actin |
| MLOC_56003 | 1 | 53 | 13 | 32 | 7 | 0.25 | 0.22 | Actin |
| MLOC_77535 | 2 | 10 | 12 | 3 | 2 | 1.20 | 0.67 |  |
| MLOC_18287 | 5 | 6 | 11 | 9 | 17 | 1.83 | 1.89 | Pore-forming toxin-like protein Hfr-2 |
| MLOC_37976 | 3 | 11 | 10 |  |  | 0.91 |  | Beta glucosidase like protein |
| MLOC_6795 | 4 | 14 | 9 | 1 | 0 | 0.64 | 0.00 | Methyltransferase type 11 |
| MLOC_69295 | 5 | 14 | 9 | 3 | 0 | 0.64 | 0.00 | Ferritin |
| MLOC_9887 | 2 | 16 | 8 |  |  | 0.50 |  | Bifunctional inhibitor/lipid-transfer, seed storage 2S albumin superfamily protein |
| MLOC_15685 | 6 | 16 | 7 | 5 | 1 | 0.44 | 0.20 | GOLD |
| MLOC_49652 | 2 | 8 | 7 |  |  | 0.88 |  | General substrate transporter/Sugar transporter |
| MLOC_67053 | 3 | 19 | 7 |  |  | 0.37 |  | Helix-loop-helix DNA-binding |
| MLOC_7780 | 1 | 17 | 7 | 4 | 2 | 0.41 | 0.50 | Malate dehydrogenase |
| MLOC_10713 | 3 | 12 | 6 | 13 | 6 | 0.50 | 0.46 | small ubiquitin-like modifier 1 |
| MLOC_10832 | 3 | 5 | 6 |  |  | 1.20 |  | THO complex subunit |
| MLOC_360 | 5 | 33 | 6 | 38 | 4 | 0.18 | 0.11 | Elongation factor 2 |
| MLOC_37449 | 5 | 22 | 6 |  |  | 0.27 |  | Heat shock protein |
